# Identification of Interphase Functions for the NIMA Kinase Involving Microtubules and the ESCRT Pathway

**DOI:** 10.1371/journal.pgen.1004248

**Published:** 2014-03-27

**Authors:** Meera Govindaraghavan, Sarah Lea McGuire Anglin, Kuo-Fang Shen, Nandini Shukla, Colin P. De Souza, Stephen A. Osmani

**Affiliations:** 1 Molecular, Cellular and Developmental Biology Program, The Ohio State University, Columbus, Ohio, United States of America; 2 Department of Molecular Genetics, The Ohio State University, Columbus, Ohio, United States of America; 3 Millsaps College, Jackson, Mississippi, United States of America; 4 Ohio State Biochemistry Program, The Ohio State University, Columbus, Ohio, United States of America; Duke University Medical Center, Durham, United States of America

## Abstract

The Never in Mitosis A (NIMA) kinase (the founding member of the Nek family of kinases) has been considered a mitotic specific kinase with nuclear restricted roles in the model fungus *Aspergillus nidulans*. By extending to *A. nidulans* the results of a synthetic lethal screen performed in *Saccharomyces cerevisiae* using the NIMA ortholog *KIN3*, we identified a conserved genetic interaction between *nimA* and genes encoding proteins of the Endosomal Sorting Complex Required for Transport (ESCRT) pathway. Absence of ESCRT pathway functions in combination with partial NIMA function causes enhanced cell growth defects, including an inability to maintain a single polarized dominant cell tip. These genetic insights suggest NIMA potentially has interphase functions in addition to its established mitotic functions at nuclei. We therefore generated endogenously GFP-tagged NIMA (NIMA-GFP) which was fully functional to follow its interphase locations using live cell spinning disc 4D confocal microscopy. During interphase some NIMA-GFP locates to the tips of rapidly growing cells and, when expressed ectopically, also locates to the tips of cytoplasmic microtubules, suggestive of non-nuclear interphase functions. In support of this, perturbation of NIMA function either by ectopic overexpression or through partial inactivation results in marked cell tip growth defects with excess NIMA-GFP promoting multiple growing cell tips. Ectopic NIMA-GFP was found to locate to the plus ends of microtubules in an EB1 dependent manner, while impairing NIMA function altered the dynamic localization of EB1 and the cytoplasmic microtubule network. Together, our genetic and cell biological analyses reveal novel non-nuclear interphase functions for NIMA involving microtubules and the ESCRT pathway for normal polarized fungal cell tip growth. These insights extend the roles of NIMA both spatially and temporally and indicate that this conserved protein kinase could help integrate cell cycle progression with polarized cell growth.

## Author Summary

All organisms have to integrate cell growth, and often the polarization of cell growth, with the rate of progression through the cell cycle. One of the most highly polarized modes of growth found in nature is displayed by the ubiquitous filamentous fungi. How the regulation of mitotic divisions is linked to polarized growth remains a mystery, but might involve mitotic regulators. One key mitotic regulator identified in the model filamentous fungus *Aspergillus nidulans* is the NIMA kinase, the founding member of the Nek family of protein kinases. This kinase is known to play mitotic specific roles within nuclei. Our genetic studies reported here reveal unexpected interactions between NIMA and six components of a pathway required for the turnover of cell membrane proteins (the ESCRT pathway) showing NIMA and ESCRT components are required together for normal polarized growth. This suggests NIMA has non-nuclear interphase functions. Further supporting this possibility, cell biological studies link NIMA to both cell tip growth and the modulation of interphase microtubule functions. Collectively the findings therefore extend the functions of the NIMA kinase beyond mitosis to include the regulation of normal polarized cell growth, suggesting this kinase might help integrate these two key aspects of growth and development.

## Introduction

All filamentous fungi, including *Aspergillus nidulans*, exhibit a characteristic extreme form of polarized growth, generating long interconnected cells termed hyphae that contain multiple nuclei in a common cytoplasm. *A. nidulans* nuclei are also known to undergo parasynchronous waves of mitosis within the common cytoplasm [1], [2]. It has long been recognized that these cycles of nuclear division alternate with periods of cell growth, indicating that there are regulatory mechanisms that underlie the integration of cell growth with mitotic regulation [3]. However, the molecular basis of how mitotic regulation in *A. nidulans* is coordinated with polarized cell growth and development is not well understood. *A. nidulans* serves as a model system not only to address basic questions in cell biology but also to study filamentous fungal biology, which is poorly understood yet highly significant, as different species of filamentous fungi can have highly harmful or beneficial effects on humans [4]. It is known that polarized growth in *A. nidulans* and other filamentous fungi is mediated by targeted secretory exocytosis at the cell apex coupled with spatially regulated endocytosis [5], [6]. A complex signaling network including cell end marker proteins, sterol-rich membrane domains and Cdc42 GTPase signaling has been proposed to mediate polarized growth at the hyphal cell tip [7]. After several rounds of mitosis coupled with polarized cell growth, *A. nidulans* cells become separated into apical and basal cell compartments by the formation of septa [8]. The nuclei in basal cell compartments are removed from the cell cycle, arresting in G1 until after the formation of a de novo cell tip (branch) at which point nuclei re-enter the cell cycle [9], [10]. Importantly however, the initiation of new cell tips is suppressed in the vicinity of actively growing cell tips, underlining a phenomenon called apical dominance [11]. Maintaining apical dominance might contribute to the fast growth rates of hyphal cells [12], since the biosynthetic capacity of many nuclei is directed towards growth at a single growing cell tip in contrast to most other cell types where only a single nucleus supports cell growth. Mutants defective in maintaining apical dominance form multiple axes of polarity near the cell apex. The identification of such mutants in *Aspergilli* has provided evidence for the genetic regulation of apical dominance [13]–[15]. Highlighting a role for the actin cytoskeleton in maintaining apical dominance, mutants of genes encoding the formin SepA, polarisome components Spa2 and Bud6, or the actinin-like protein AcnA, lead to the generation of abnormal secondary polarity axes in the vicinity of the cell tip [16]–[18]. In addition, studies have also indicated that cell-tip-localized calcium signaling and reactive oxygen species function in ensuring the dominant growth of a single hyphal cell tip [19]–[21]. Although apical dominance is a recognized feature of filamentous fungal growth, more remains to be learned, especially with regard to the regulation of de novo cell tip formation and how this is integrated with cell cycle regulation.

The NIMA kinase was identified in a forward genetic screen performed in *A. nidulans* and is essential for entry into mitosis, along with the mitotic Cdk1 kinase [22]–[24]. NIMA is the founding member of the NIMA related kinase (Nek) family which has conserved mitotic functions in all eukaryotic cells, including humans and plants [25]. Human Neks have also been shown to regulate other key cellular processes such as the DNA damage response and ciliogenesis [25], [26]. Underlining the conservation of NIMA function, overexpression of NIMA can promote mitotic events not only in *A. nidulans* but also strikingly in fission yeast, *Xenopus*, and human cells [27], [28]. In *A. nidulans*, temperature sensitive *nimA* mutants arrest in late G2 at the restrictive temperature [23], [29]. A genetic screen aimed at identifying extragenic suppressor mutations of a *nimA* mutant allele led to the identification of two genes encoding proteins of the nuclear pore complex (NPC) [30], [31], suggesting that NIMA might regulate the G2-M transition by modifying the function of the NPCs, thereby allowing tubulin and other mitotic regulators to enter the nucleus. Indeed, expression of NIMA can promote the mitotic dispersal of peripheral NPC proteins, even out of cell cycle phase [32], implicating NIMA in regulating nuclear envelope permeability at mitotic entry. Importantly, recent evidence indicates that this function of NIMA is conserved among the mammalian NIMA-related kinases Nek6 and Nek7 [33]. NIMA overexpression can promote chromatin condensation and the phosphorylation of histone H3 S10, a universal mark of mitotic chromatin, even out of cell cycle phase [34]. Analysis of NIMA-interacting proteins identified from a yeast two hybrid interaction screen point to a role for NIMA in other mitotic processes such as the regulation of astral microtubules and nuclear envelope dynamics [35], [36].

Although the inactivation of NIMA using temperature sensitive mutant alleles at the restrictive temperature prevents mitotic entry, it allows short term polarized growth to the extent that can be supported by a single nucleus. These results, taken together with studies showing that NIMA locates to distinct nuclear structures during mitosis [32], [34] had led to the idea that the functions of NIMA are exclusively at the nucleus during mitosis. Our studies described here, however, support a previously unrecognized cytoplasmic function for NIMA in regulating cell growth during interphase. Taken together with the requirement for NIMA in mitosis, this suggests that NIMA has the potential to help integrate cell growth and development with mitotic regulation.

## Results

### Identification of conserved genes that are essential when *nimA* is partially inactivated

Although *nimA* is an essential gene, the *S. cerevisiae* orthologue *KIN3* is non-essential [37]–[39]. Therefore, a *KIN3* deleted strain was used to perform a genetic screen to identify non-essential gene deletions that are synthetically lethal in the absence of *KIN3*. Since *KIN3* does not appear to have an essential mitotic function, we reasoned this approach might identify conserved genetically interacting partners potentially involved in non-mitotic functions of *KIN3* and hence, by extension, NIMA. Eighty-three genes were identified as being potentially synthetic lethal or synthetic sick with *ΔKIN3* in the screen. Of these, eleven genes were confirmed by tetrad analysis as having synthetic interactions with the *KIN3* deletion (Table 1). Further details of this screen are to be published elsewhere.

**Table 1:** *A. nidulans* orthologues of genes that are synthetically lethal with Δ*kin3* in *S. cerevisiae*.

*S. cerevisiae* Gene Name	A. nidulans		E-value^c^	Function in *S. cerevisiae*	Genetically interacts with *nimA7* in *A.* *nidulans* d
	Gene^a^	Deletion phenotype^b^			
Zuo1	AN7143	Slow growth	1.0e-83	Cytosolic ribosome associated chaperone for nascent polypeptide chains	No
Ssz1	AN4616 (Ssz1 [95], [96])	Slow growth	5.0e-122	Hsp70 protein that interacts with Zuo1 to a form a ribosome-associated complex	No
Mph1	AN10063	Viable	3.0e-90	Member of DEAH family of helicases; functions in an error-free DNA damage bypass pathway	No
Bud14	AN1099 (TeaC [97])	Viable	4.0e-11	Protein involved in bud-site selection	No
Dot1	AN0091	Viable	7.0e-44	Nucleosomal histone H3-K79 methyl transferase	No
Hsl7	AN0134 (RmtC [98])	Viable	1.0e-67	Protein arginine N-methyltransferase that exhibits septin and Hsl1p dependent bud neck localization	No
Ypt7	AN0089 (AvaB [99])	Viable	1.0e-77	GTPase of the rab family required for homotypic fusion event in vacuole inheritance and endosome-endosome fusion.	No
Swd1	AN0808	Sick	5.0e-68	Subunit of the Set1C (COMPASS) complex which methylates histone H3 at K4.	Yes
Vps23	AN2521 (Vps23[51])	Very sick	2.0e-10	Component of the ESCRT-I complex; involved in ubiquitin-dependent sorting of proteins into endosomes	Yes
Vps25	AN11722	Very sick	2.7e+00	Component of the ESCRT-II complex; involved in ubiquitin-dependent sorting of proteins into endosomes	Yes

a Designation given by the *A. nidulans* Genome database.

b Refers to the growth phenotype of the deletion strains on complete media (Figure 1, Supplementary Figure S1, and data not shown).

c Values obtained by BLASTp using the *S. cerevisiae* protein to query the *A. nidulans* Genome database at AspGD.

d This study.

To test whether the genetic interactions identified in *S. cerevisiae* were conserved in *A. nidulans*, we identified the *A. nidulans* orthologues of the *S. cerevisiae* genes that are synthetic lethal with *KIN3* using BLASTp searches at the Aspergillus Comparative Database of the Broad Institute and the Aspergillus Genome Database (www.aspgd.org, [40]) (Table 1). We deleted each of these genes in a strain WT for *nimA* and in a strain carrying the *nimA7* temperature sensitive mutation. The phenotype of the gene deletions in NIMA^+^ backgrounds is indicated in Table 1. Genetic interactions between these gene deletions and *nimA7* were identified by comparing the colony growth of the double mutants to the single mutants at the semi-permissive temperature for *nimA7*. This analysis revealed that many of the deletions did not cause synthetic lethality with *nimA7* but three did display synthetic genetic interactions (Table 1, Figure 1A, B, Figure S1 in supporting material and data not shown) that are shared by *ΔKIN3* in *S. cerevisiae*. Two of these represent mutations of genes encoding the orthologues of Vps23p and Vps25p, which are both components of the membrane trafficking ESCRT pathway involved in protein degradation via the formation of multi-vesicular bodies as well as in cytokinesis and retroviral budding [41]–[49]. Our analysis of the genetic interactions of *nimA7* with *ΔAn-vps23, ΔAn-vps25* and other components of the ESCRT pathway is presented here. The analysis of the genetic interaction between *nimA7* and *ΔAn-swd1* is the focus of a separate study.

**Figure 1:**
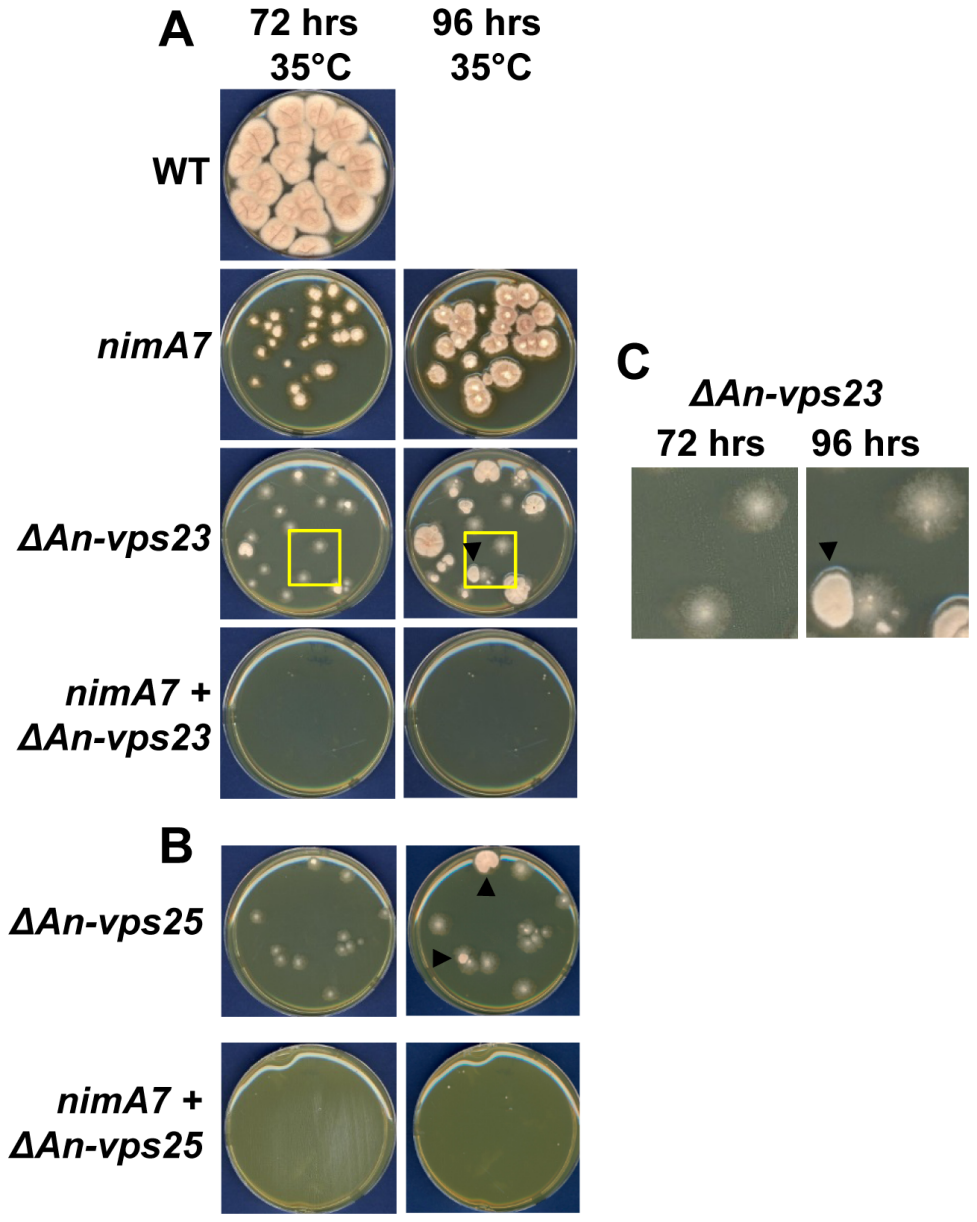
*An-vps23* and *An-vps25* are essential when *nimA* is partially inhibited. (A and B) At 35°C, a semi-permissive temperature for *nimA7*, absence of *An-vps23* or *An-vps25* is poorly tolerated in combination with the temperature sensitive *nimA7* allele, resulting in the absence of any colony growth even after 96 hours of incubation in the double mutant *nimA7* + *ΔAn-vps23* and *nimA7* + *ΔAn-vps25* strains. Black arrowheads mark Δ*An-vps23* and Δ*An-vps25* colonies that exhibit the presence of suppressor mutations at 96 hours but not at 72 hours. (C) The inset in (A) has been magnified to show the emergence of colonies carrying suppressor mutations from one colony and not from the adjacent one. Strains: WT  =  R153, *nimA7*  =  MG44, *ΔAn-vps23*  =  MGH21, *ΔAn-vps25*  =  MGH26, *nimA7* + *ΔAn-vps23*  =  MGH17, *nimA7* + *ΔAn-vps25*  =  MGH14.

### Deletion of *An-vps23* or *An-vps25* results in the formation of heterokaryons indicating they are essential for normal growth

In *A. nidulans*, during heterokaryon rescue [50], deletion of essential genes (or genes required for normal growth) leads to the formation of heterokaryons which carry both WT nuclei lacking the *pyrG^AF^* nutritional marker of the deletion cassette, as well as nuclei lacking the essential gene but which carry the *pyrG^AF^* nutritional marker. The presence of two genetically different nuclei in the heterokaryon is confirmed using diagnostic PCR. During asexual spore development, heterokaryons, like haploid homokaryon colonies, form uninucleate spores (conidia). The conidia from heterokaryons can therefore carry either parental nuclei or nuclei with the target gene deleted. The phenotypes caused by the lack of the essential gene can be studied by germinating the conidia from the heterokaryon colonies on selective media for *pyrG^+^*. We found that deletion of *An-vps23* or *An-vps25* results in the formation of heterokaryons (Figure S2 in supporting material). When conidia from *ΔAn-vps23* or *ΔAn-vps25* heterokaryons are spread on *pyrG^+^* selective plates to allow single colony formation, only small poorly conidiating colonies are able to form (Figure 1), indicating that *An-vps23* and *An-vps25* are not completely essential for growth although the deletion alleles can only be effectively propagated through heterokaryons.

The colonies formed by *An-vps23* or *An-vps25* deleted spores often give rise to one or more spontaneously occurring better growing sectors (Figure 1A, arrowheads). Figure 1C shows a magnified image of the same *ΔAn-vps23* colonies at 72 and at 96 hours of growth at 35°C. One colony does not show the generation of a faster growing sector, grows slowly and conidiates poorly, while the other displays the emergence of faster growing sectors that are distinct in appearance and show better conidiation (arrowhead). The data indicate that the growth defects caused by deletion of *An-vps23* or *An-vps25* exert a selective pressure for spontaneous suppressor mutations to arise that can partially rescue the growth defects of the null alleles. Consistent with this possibility, the generation of the faster growing sectors was found to depend on some degree of growth of the mutant colonies to allow the suppressor mutations to be generated. While our study was in progress similar spontaneous suppressor mutations were independently identified and were defined to be present in at least two cation tolerance genes, one of which is the transcription factor *sltA* [51]. Propagating the *ΔAn-vps23* and *ΔAn-vps25* alleles in heterokaryons ensured we did not select for suppressor mutations in our strains before phenotypic analysis.

### *An-vps23* and *An-vps25* are essential when *nimA* is partially inactivated

To test for synthetic genetic interaction between *An-vps23* or *An-vps25* and *nimA*, the colony growth of conidia isolated from the double mutants *ΔAn-vps23 + nimA7* and *ΔAn-vps25 + nimA7* was compared with the growth of the single mutants. At the semi-permissive temperature of 35°C, strains carrying the *nimA7* allele form restricted colonies as compared to WT colonies grown at the same temperature (Figure 1). Conidia from strains deleted for *An-vps23* or *An-vps25* also form small aconidial colonies that eventually generate some suppressor fans as described above. In contrast, conidia from double mutant cells are unable to form colonies even after 96 hours of incubation at 35°C (Figure 1A, B). This growth assay indicates that when *nimA* is partially inactivated, *An-vps23* and *An-vps25* are essential for growth.

### Deletion of *An-vps23* or *An-vps25* modifies the terminal phenotype of cells lacking *nimA* function

**Figure 2:**
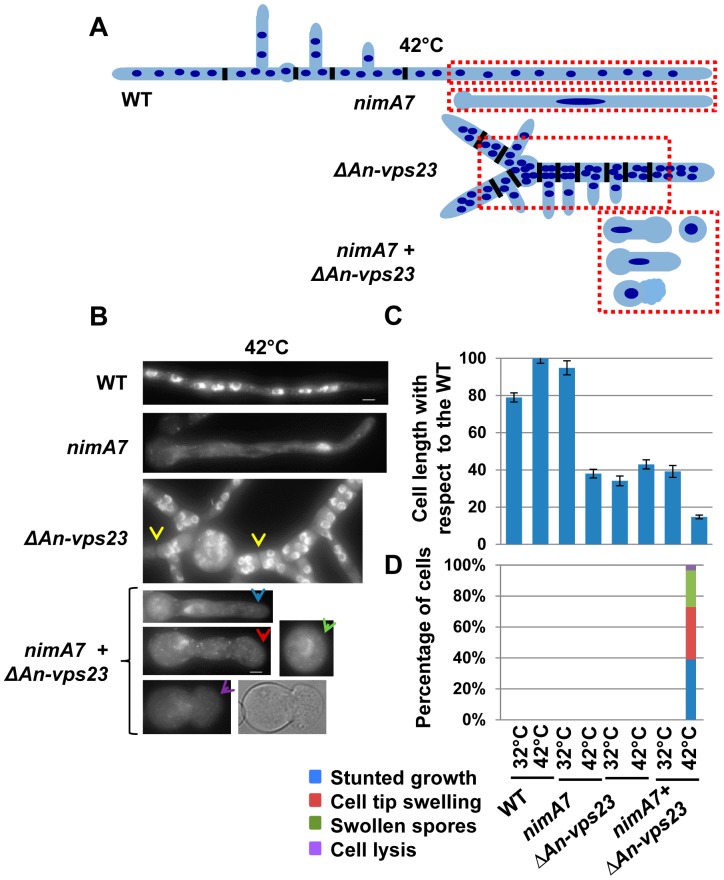
Deletion of *An-vps23* modifies the terminal phenotype of cells lacking NIMA function. (A) A schematic representation of the cell morphological phenotypes of WT, *nimA7*, *ΔAn-vps23* and *nimA7 + ΔAn-vps23* strains. The red dotted rectangles mark the region of the cell in each strain that is depicted in (B). (B) Representative images of the indicated strains. Closely spaced septa (as identified by bright field imaging) in Δ*An-vps23* cells are marked by yellow arrows. (C) The double mutants show a higher, statistically significant growth defect at 42°C, the restrictive temperature for the *nimA7* allele (blue arrow in B) compared to either single mutant (p<0.001). (D) The double mutants display novel phenotypes not seen in either single mutant at 42°C, such as defects in germtube emergence (green arrowhead), cell tip swelling (red arrowhead), and cell lysis (purple arrowhead). Bar, 5 μm. WT  =  R153, *nimA7*  =  MG44, *ΔAn-vps23*  =  MGH21, *nimA7* + *ΔAn-vps23*  =  MGH19.

Our data indicate that partial loss of *nimA* function is not tolerated when combined with the absence of *An-vps23* or *An-vps25*. We therefore determined if the terminal phenotype of the double mutants at the fully restrictive temperature would be more severe than the terminal phenotypes of either single mutant. To test this we germinated conidia from WT, Δ*An-vps23*, Δ*An-vps25*, and *nimA7* single mutants and the *ΔAn-vps23 + nimA7* and *ΔAn-vps25 + nimA7* double mutants at the restrictive temperature for *nimA7* (42°C). The cells were fixed and stained with DAPI to visualize nuclei and analyzed by microscopy. WT cells grown at 42°C exhibited normal growth morphology, generating multinucleated cells as shown by the representative image in Figure 2B and the illustration in Figure 2A. At restrictive temperature, *nimA7* cells were defective in initiating mitosis and arrested with a single interphase nucleus. However, they could undergo short term growth to the point that can be supported by a single nucleus (Figure 2A, B). Unlike WT cells, *An-vps23* deleted cells were wider such that nuclei could be found adjacent to each other across the width of the germtube (Figure 2A, B). *An-vps23* deleted cells also noticeably showed closely spaced septa and branches as identified by brightfield microscopy and indicated by yellow arrows in Figure 2B. These growth defects indicate that *An-vps23* is required for normal cell growth morphology. Importantly, consistent with the synthetic lethal interaction between *nimA* and *An-vps23*, we found that the double *ΔAn-vps23 + nimA7* mutant exhibited more severe growth defects when compared to either single mutant (Figure 2C, Figure 2B, blue arrow). Moreover, the double mutants also showed novel cell growth defects not seen in either single mutant (Figure 2D), including cell tip swelling (Figure 2B, red arrow), defects in the initiation of polarized growth (Figure 2B, green arrow), and cell lysis (Figure 2B, purple arrow). *An-vps25* deleted cells and *ΔAn-vps25 + nimA7* double mutants showed similar cell growth defects as their *An-vps23* counterparts (data not shown), indicating a common basis for the synthetic genetic interaction between *An-vps23* and *An-vps25* with *nimA7*. To define if the modified phenotypes are a result of the cells containing a single G2 nucleus or are specific to NIMA we used a different mutation to cause G2 arrest. We looked at the growth defects of *ΔAn-vps23* in combination with inactivation of the Cdc25 phosphatase, which is required for mitotic activation of Cdk1 and hence mitotic entry (Cdc25 is called *nimT* in *A. nidulans* [52]). This study revealed that the temperature sensitive *nimT23* allele does not cause arrest in G2 as effectively as *nimA7* at restrictive temperatures. We could not therefore determine if the modified growth defects seen in the Δ*vps + nimA7* double mutants are specific to NIMA inactivation or a consequence of arrest in G2 with a single nucleus.

### *nimA* functions in concert with *An-vps23* to maintain tip cell apical dominance

**Figure 3:**
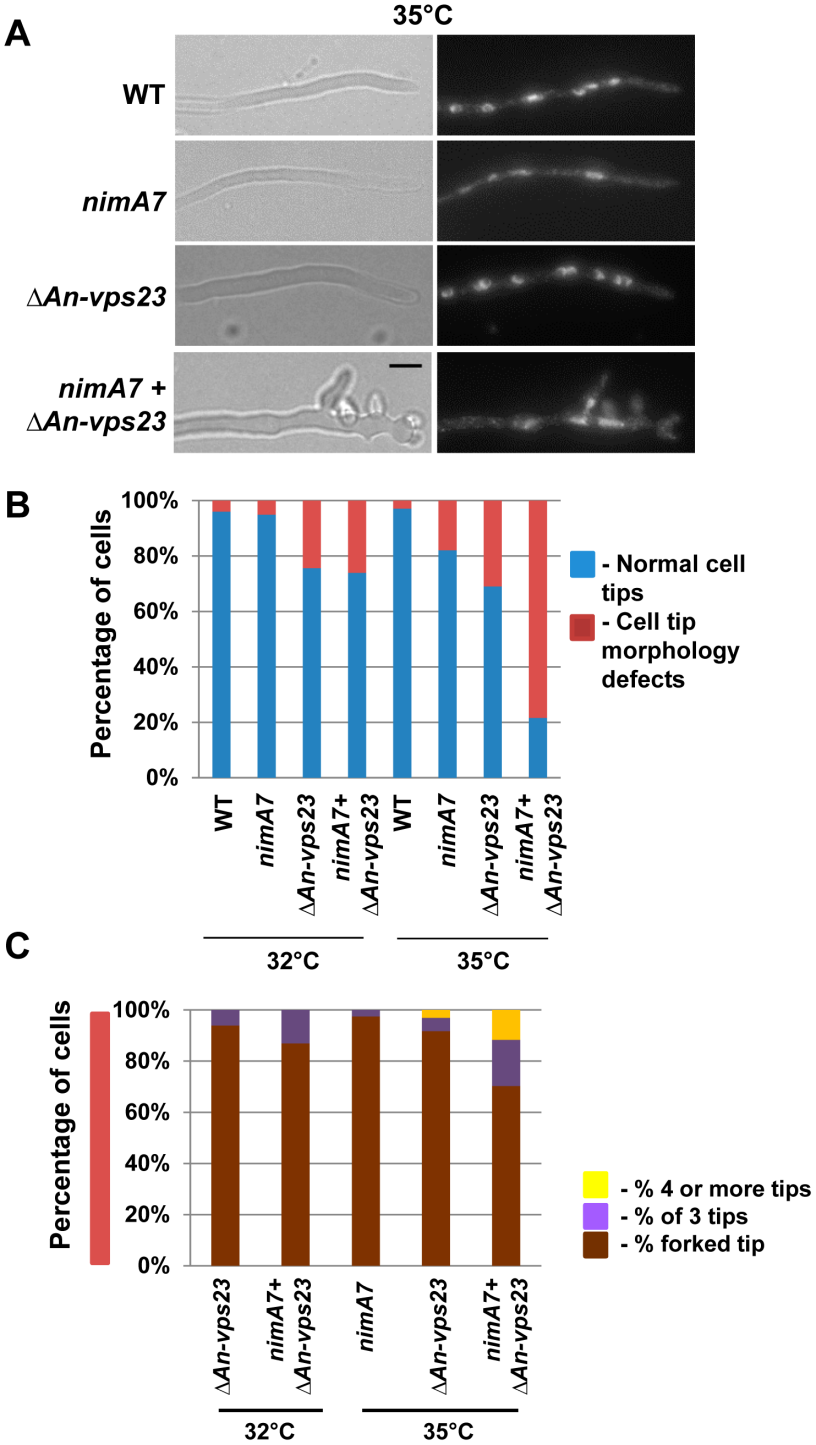
NIMA functions in concert with An-Vps23 to maintain cell tip apical dominance. (A) Representative images show enhanced defects in maintaining apical dominance in the double mutant (*nimA7* + *ΔAn-vps23*) compared to either single mutant. (B) Quantitation of cell tip morphology defects (loss of apical dominance and cell tip swelling). (C) Quantitation of number of additional tips at the cell apex as a measure of the severity of tip morphology defect in the double mutant. Only cells that show appreciable defects in tip morphology as seen in (B) are represented. Strains: WT  =  R153, *nimA7*  =  MG71, *ΔAn-vps23*  =  MGH21, *nimA7* + *ΔAn-vps23*  =  MGH19.

To gain insights into the basis for the synthetic genetic interaction between *An-vps23* and *nimA*, we sought to determine the cellular phenotype of the double mutants in comparison with the single mutants at the semi-permissive temperature. WT strains, *ΔAn-vps23* or *nimA7* single mutant strains and double mutants were grown at the semi-permissive temperature, fixed and stained with DAPI to visualize nuclei. At the permissive temperature for *nimA7* allele, (32°C) almost all WT and *nimA7* cells had normal cell tip morphology (Figure 3B). However, a proportion of cells with partial *nimA7* function (*nimA7* cells at 35°C) exhibited cell growth defects including dichotomous cell tips and cell tip swelling, when compared to the WT cells at 35°C (Figure 3). Among Δ*An-vps23* cells, a fraction exhibited defects in maintaining apical dominance, a phenotype that was largely independent of temperature (Figure 3A, B). Interestingly, the population of double mutant cells that exhibited defects in maintaining a single growing cell tip was greater than the sum of the single mutant populations showing defective cell tip morphology, indicating the enhanced nature of the genetic interaction (Figure 3A, B). Importantly, this phenotype was temperature-dependent and only seen at 35°C, the temperature at which *nimA* is partially inactivated (Figure 3B). In addition to an increase in the percentage of double mutants displaying a breakdown of apical dominance, double mutant cells also showed more severe forms of the phenotype (Figure 3C). For instance, around 10% of double mutant cells had three growing tips at the cell apex and around 12% had four or more tips at the cell apex, compared to a far smaller percentage of cells showing either phenotype in the single mutants.

### The function of multiple ESCRT pathway components is required for colony growth when *nimA* is partially inactivated

**Figure 4:**
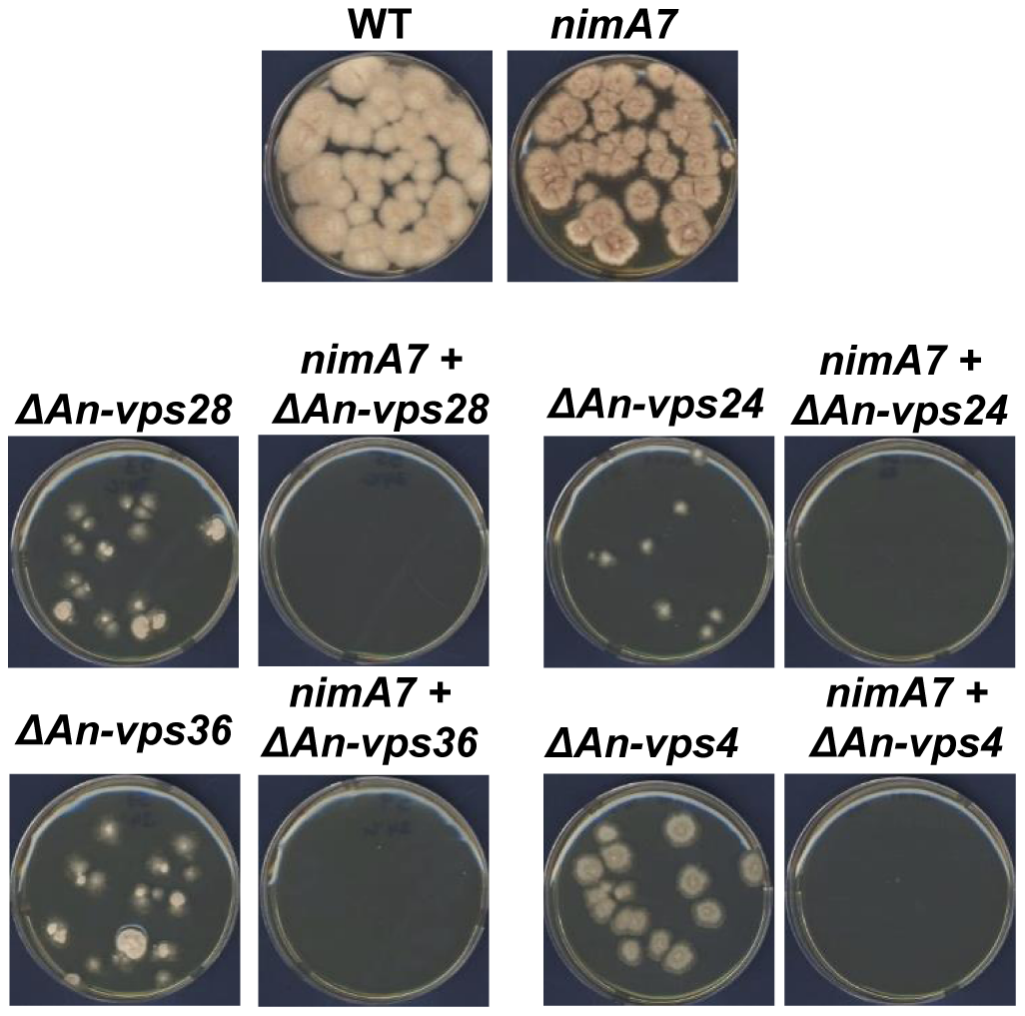
Multiple ESCRT pathway genes become essential when NIMA function is partially reduced. Colonies were grown from spores of the indicated genotypes for 96-permissive temperature for *nimA7* (35°C), the double mutants lacking either *An-vps28*, *An-vps36*, *An-vps24*, or An-*vps4* do not show any colony formation indicating a synthetic lethal interaction between *nimA7* and these deletion alleles. WT  =  R153, *nimA7*  =  MG71, *ΔAn-vps28*  =  MGH53, *nimA7* + *ΔAn-vps28*  =  MGH55, *ΔAn-vps24*  =  MGH49, *nimA7* + *ΔAn-vps24*  =  MGH51, *ΔAn-vps36*  =  MGH57, *nimA7* +*ΔAn-vps36*  =  MGH59, *ΔAn-vps4*  =  MGH45, *nimA7* + *ΔAn-vps4*  =  MGH47.

The synthetic genetic interaction between *An-vps23* (component of the ESCRT-I complex) and *An-vps25* (component of the ESCRT-II complex) with *nimA7* could be specific to the function of these two genes or due to an essential role for the ESCRT pathway in cells with partial *nimA* function. To address this, we generated strains deleted for *An-vps28* (component of ESCRT I complex), *An-vps36* (component of ESCRT II complex), *An-vps24* (component of ESCRT III complex) and *An-vps4* (an AAA-ATPase which interacts with ESCRT III complex proteins) in WT or *nimA7* strain backgrounds [41], [43], [44], [53], [54]. The deletion of each of the *An-vps* genes in combination with partial *nimA* function resulted in synthetic growth defects to at least the same extent as *ΔAn-vps23 + nimA7* or *ΔAn-vps25 + nimA7* (Figure 4).

As previously reported [51], we also observed spontaneous suppressor mutations arise in the *An-vps28*, *An-vps36*, and *An-vps24* deleted strains. To address the possibility that the spontaneous *vps* suppressor mutations might also suppress the synthetic genetic interactions with *nimA7*, we allowed suppressor mutations to arise from *nimA7* + Δ*vps36* colonies. This was achieved by allowing colony growth of haploid *nimA7* + Δ*vps36* strains at permissive temperature until revertant colonies arose. Conidia collected from such revertants were then re-tested for synthetic growth defects at 35°C. This revealed that the spontaneous suppressor mutations suppress the interaction between *nimA7* and *Δvps36* (Figure S3 in supplementary material). However, in contrast to the other *vps* gene deletions, the Δ*An-vps4* strain notably formed larger colonies and did not pick up suppressor mutations but revealed the most marked synthetic lethality in combination with *nimA7* (Figure 4). Collectively the genetic analysis indicates that the function of complexes acting at different points of the ESCRT pathway becomes essential when *nimA* is partially inactivated.

### The NIMA kinase locates to growing cell tips

**Figure 5:**
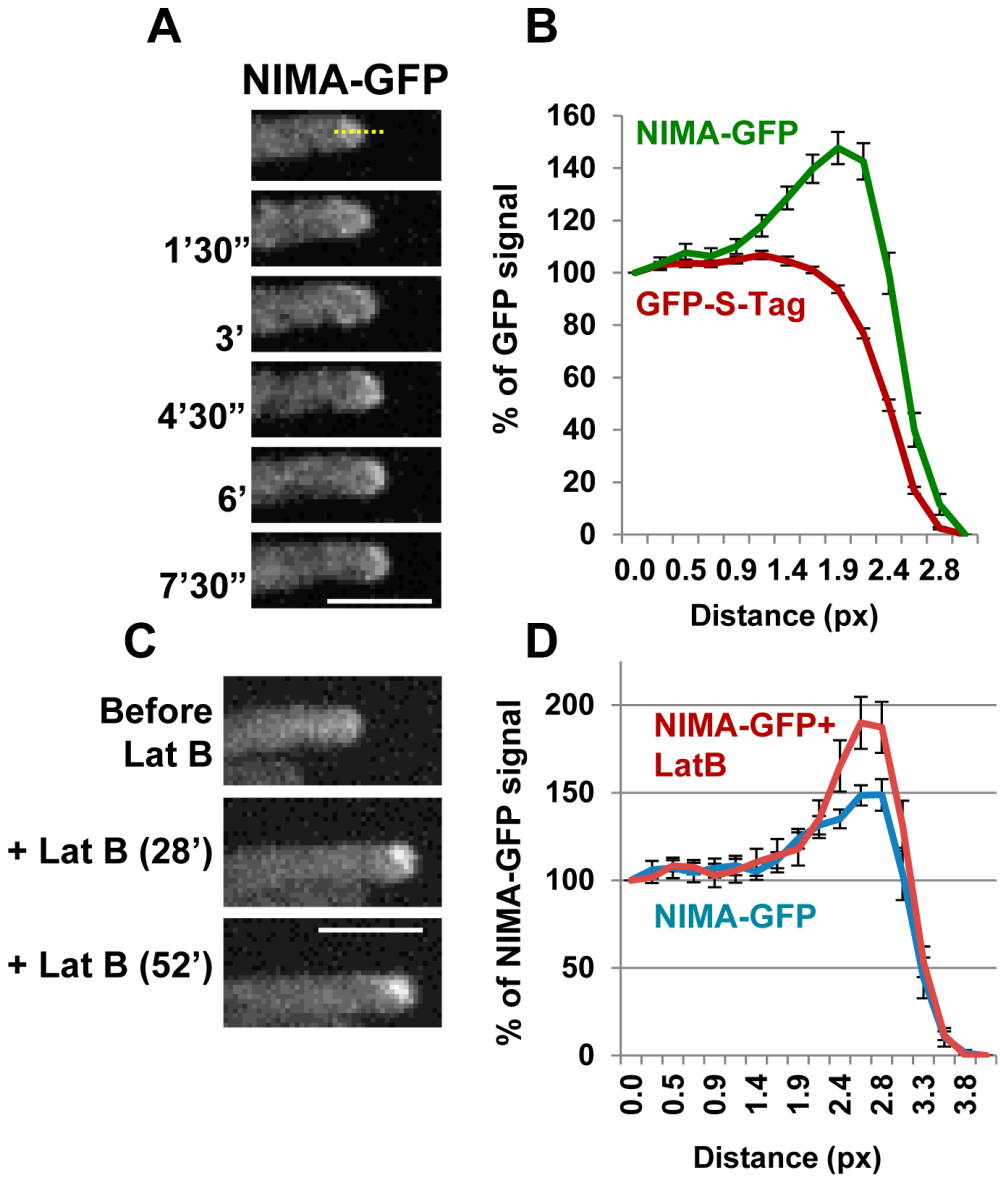
The NIMA kinase localizes to growing cell tips. (A) NIMA-GFP exhibits a dome-shaped localization at the tips of growing hyphal cells (strain: KF45). See also Movie 1 in supporting material. (B) The average percentage signal intensity for NIMA-GFP (KF45) or GFP-S-Tag (CDS1068) along a ROI drawn across the cell tip. The background fluorescence signal from untagged cells was subtracted and the signal was normalized to the signal inside the cell (taking the first value on the X axis as 100%). (C) Increase in NIMA-GFP signal seen at 70% of the cell tips after depolymerization of actin by latrunculin B (strain: KF45). (D) Quantitation of the percentage of NIMA-GFP signal in cells that exhibited an increase in NIMA at the cell tips after treatment with latrunculin B (n = 19). Normalization of GFP signal was done as in (B). Bars, 5 μm.

We next sought to determine whether the localization of NIMA might provide insights into its functions during cell growth, as suggested by the genetic analysis above, not involving its established mitotic nuclear functions. Strains carrying the replacement of NIMA with functional NIMA-GFP at the endogenous locus were generated and analyzed using spinning disc 4D live cell confocal microscopy. In addition to confirming the previously defined mitotic locations of ectopically expressed NIMA [34], these studies revealed an additional unexpected location for NIMA-GFP at cell tips. Figure 5A shows that a low level of NIMA-GFP protein is present in a dome-shaped location at the growing cell tip (also Movie 1 in supporting material). Confirming that it is the NIMA portion of the NIMA-GFP chimera that is responsible for the cell tip location, cytoplasmic GFP tagged with the S-Tag peptide (De Souza et al., In Press) did not show the tip high distribution as revealed by the respective pixel intensity line profiles (Figure 5B). Intracellular transport towards the cell tip in *A. nidulans* is mediated by both microtubules and actin based systems [7]. We sought to determine whether microtubules or actin were necessary for locating NIMA to the cell tip. NIMA-GFP is able to locate both to cell tips and to newly emerging branch tips when microtubules are depolymerized with benomyl [55] (data not shown). When actin was depolymerized with latrunculin B [5] 70% of the cell tips showed transient increases of NIMA-GFP near the cell tips at a level higher than before drug addition (Figure 5C and D). However, the absence of ESCRT pathway function (*ΔAn-vps23*) did not obviously affect the cell tip localization of NIMA (Figure S4A in supporting material). In addition, the levels of NIMA-GFP at the cell tip were seen to diminish during mitosis, when NIMA distinctly locates to the nucleus (Figure S4B in supporting material) [32], [56].

### Overexpression of NIMA in rapidly growing hyphal cells promotes the development of multiple growth tips at the cell apex

**Figure 6:**
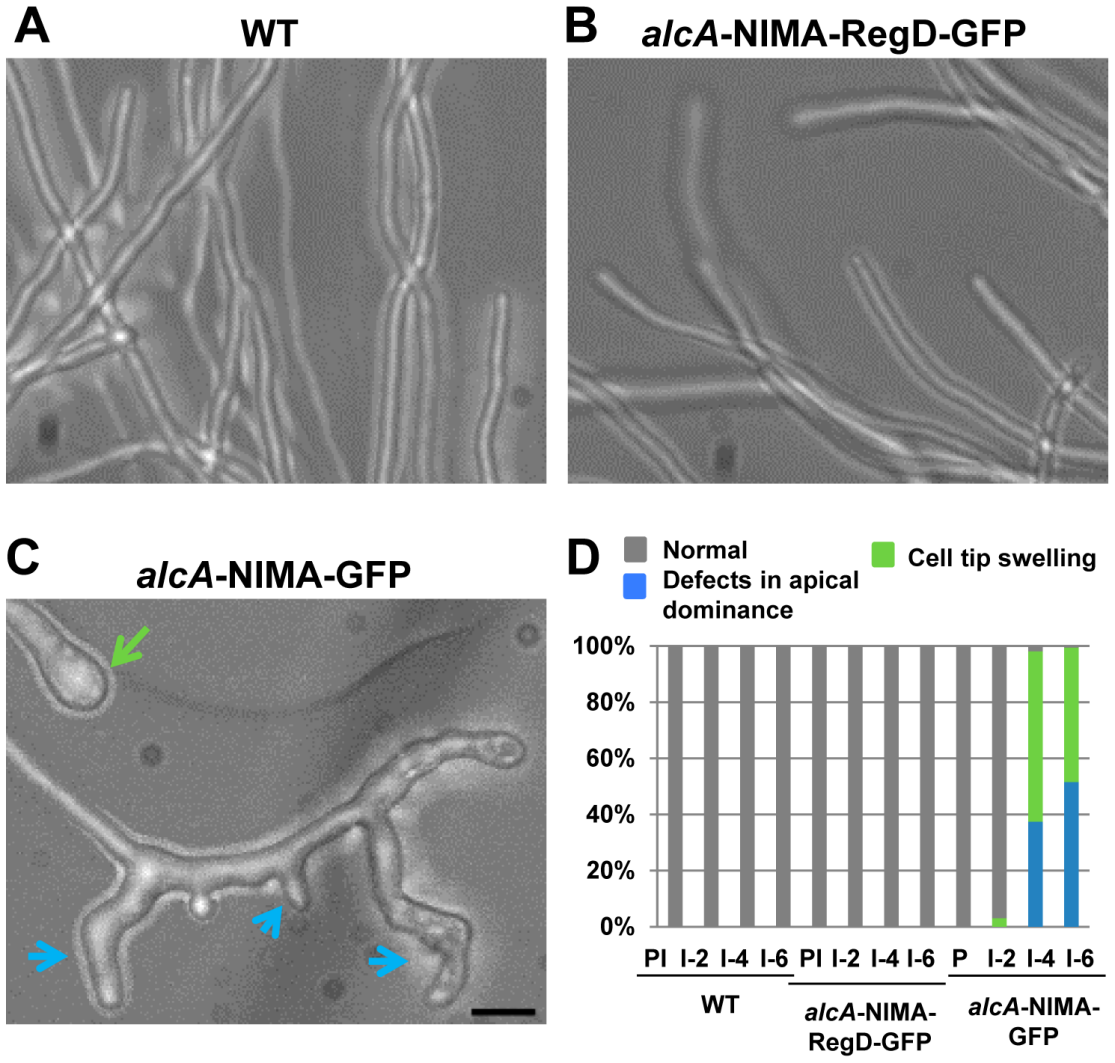
Induction of ectopic NIMA results in defects in tip cell morphology. (A) Hyphae of wildtype cells or (B) cells carrying *alcA* driven expression of full length NIMA (*alcA*::NIMA-GFP, strain: CDS683) or (C) the C-terminal regulatory domain (*alcA*::NIMA-RegD-GFP, strain: CDS131) were grown under non-inducing conditions and expression of the respective NIMA constructs was induced by the addition of threonine. Representative images of cells after 6 hours of induction are shown. Breakdown of apical dominance and cell tip swelling in NIMA-GFP expressing cells are indicated by blue and green arrows respectively. (D) Quantitation of tip growth defects. PI  =  Pre-induction, I  =  *alcA* induced for 2, 4 or 6 hours as indicated. Bar, 5 μm.

The genetic analysis and interphase cytoplasmic location described above suggests NIMA might have a role in regulating cell tip growth. To investigate this possibility, WT cells or cells carrying either NIMA-GFP or a truncated version of NIMA comprising the C-terminal regulatory domain (NIMA-RegD-GFP) under the regulatable *alcA* promoter were grown to the rapidly growing hyphal stage in liquid flask cultures. The expression of the NIMA constructs was then induced by addition of threonine, an inducer of *alcA* [57]. In WT cells, before or after addition of threonine, and before induction of *alcA* in cells carrying ectopic NIMA-GFP or NIMA-RegD-GFP, cell tips exhibited normal uniform morphology (Figure 6). Induction of NIMA-GFP promoted a dramatic modification of tip cell growth which created multiple branching events, revealing a dramatic breakdown of apical dominance (Figure 6C, blue arrows). Some NIMA-GFP-expressing cells also showed marked cell tip swelling (Figure 6C, green arrow and D). Quantitation of these phenotypes (Figure 6D) shows that excess NIMA-GFP promotes swelling and branching of apical cell tips with high penetrance. Cells expressing NIMA-RegD-GFP under similar conditions resembled WT cells (Figure 6B and D) indicating full length NIMA-GFP is required to promote misregulation of cell growth. The expression of NIMA-GFP results in more severe defects in colony growth and conidiation compared to the expression of NIMA-RegD-GFP (Figure S5A in supporting material), consistent with their relative abilities to affect cell tip growth morphology.

The data indicate that, in addition to its well established roles in mitotic regulation, NIMA has the potential to play roles during interphase involving regulation of cell tip growth.

### Ectopic NIMA-GFP displays a microtubule dependent localization to dynamic cytoplasmic comets

**Figure 7:**
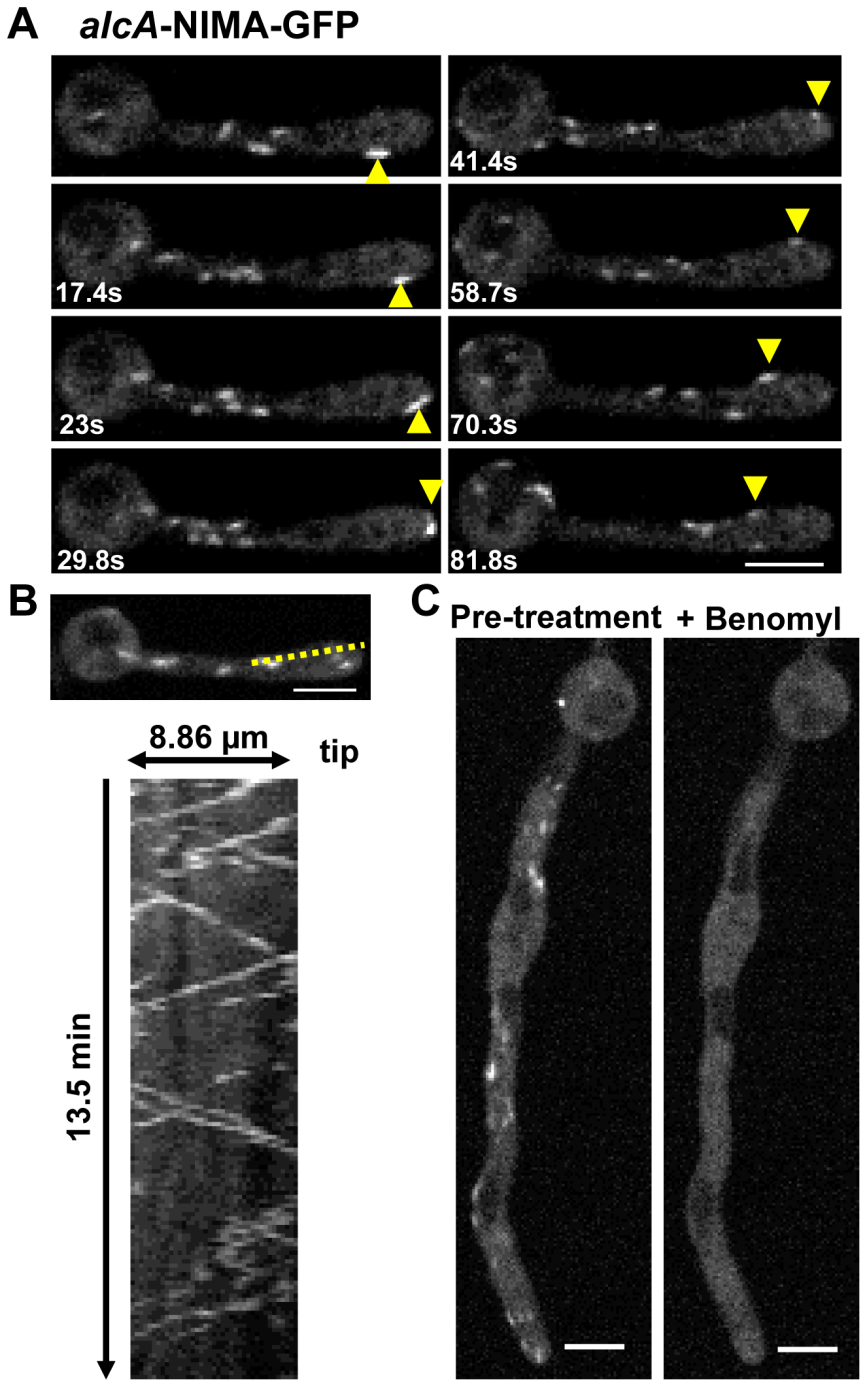
Ectopic NIMA-GFP displays a distinctive microtubule-dependent localization. (A) Ectopic NIMA-GFP (Strain: CDS683) forms dynamic cytoplasmic comets, some of which move to and around the cell tip (yellow arrowheads) when *alcA* driven NIMA expression is induced by germinating cells in glycerol. See also Movie 2 in supporting material. (B) Ectopic NIMA-GFP comets often exhibit bi-directional movement near the cell tip as revealed in the kymograph. (C) Upon addition of the microtubule poison benomyl, ectopic NIMA-GFP disperses from the comets throughout the cytoplasm. Bars, 5 μm.

Ectopic NIMA-GFP was found to locate to dynamic cytoplasmic comets (Figure 7A and B, Movie 2 in supporting material), which exhibit an average moving rate of 9+/−6 μm/min (n = 104). This rate is similar to the average polymerization rate of microtubules in *A. nidulans* [58]–[60], suggesting that the NIMA-GFP comets might be associated with the growing ends of microtubules. After addition of the microtubule depolymerizing drug benomyl, NIMA-GFP comets immediately dispersed (Figure 7C) showing the comets are dependent upon polymerizing microtubules for their formation. The rapid movement of the NIMA-GFP comets and their immediate dispersal in benomyl is similar to the behavior of the microtubule plus end binding protein EB1 in *A. nidulans* under identical conditions (data not shown). Notably however, some NIMA-GFP comets exhibited bidirectional movement near the cell tips (82% cells, Figure 7A and B), a behavior not seen for EB1 in WT cells. The montage in Figure 7A shows an example of a cell in which it was possible to track the movement of a NIMA-GFP comet towards and then around the tip as indicated by the arrowheads. The kymograph generated from Movie 2 in supporting material using the ROI indicated in Figure 7B reveals bidirectional movement of NIMA-GFP comets near the cell tip. 54% of these cells also displayed a swollen tip morphology (Figure 7A) or swelling along the germtube (data not shown), indicating that germination of cells with extra NIMA-GFP expression affects cell growth (Figure S5B in supporting material) as well as microtubule dynamics.

### Ectopic NIMA-GFP locates to the polymerizing ends of microtubules in an EB1-dependent manner

**Figure 8:**
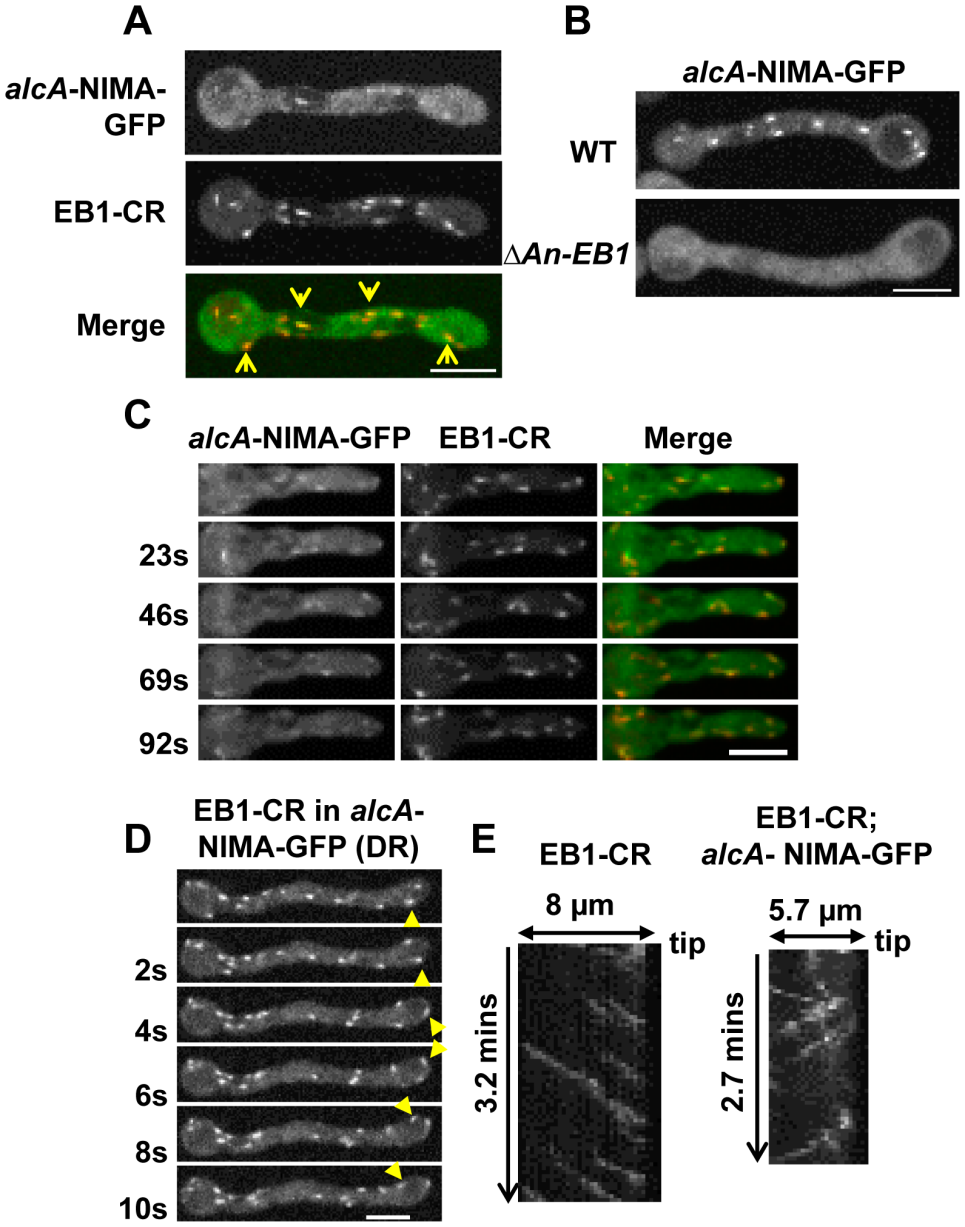
Ectopic NIMA-GFP locates to the plus ends of microtubules in an EB1 dependent manner. (A) When *alcA* driven NIMA expression is induced by germinating cells in the presence of glycerol, ectopic NIMA-GFP comets locate to microtubule plus ends defined by their colocalizaton with EB1-CR (arrows, strain: MG385). (B) The location of ectopic NIMA-GFP to microtubule plus ends is abolished in strains deleted for EB1. Strains: WT  =  MG409, ΔEB1  =  MG410. (C) Ectopic NIMA-GFP comets colocalize with EB1 through time (strain: MG385). See also Movie 3 in supporting material. (D) Expression of ectopic NIMA-GFP promotes EB1 comet movement to and then around cell tips (tracked by the yellow arrowhead, strain: MG385). See also Movie 4 in supporting material. (E) Kymographs showing only parallel traces of EB1 movement towards the cell tip when NIMA is not expressed compared to conditions when NIMA is expressed which causes EB1 comets to move both towards and away from the cell tip (strain: MG385). Bars, 5 μm.

**Figure 9:**
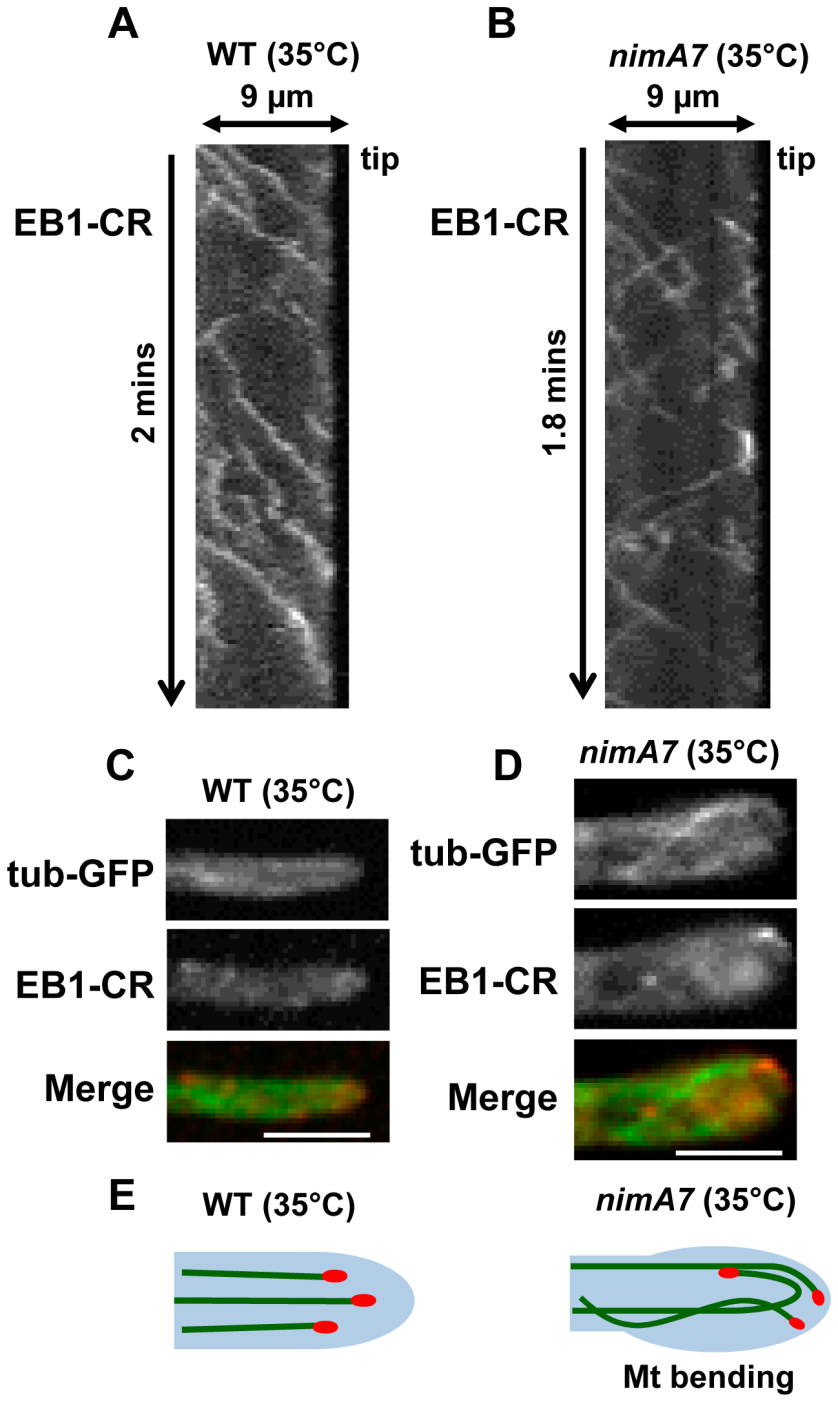
Partial inhibition of NIMA results in alteration of microtubule dynamics and EB1 behavior. (A) Kymograph showing that in WT cells (strain: MG395), EB1-CR typically moves unidirectionally towards the cell tip. (B) When NIMA function is partially impaired (strain: MG397), the movement of EB1-CR is altered to give instances of EB1 comets moving away from the cell tip. (D) Microtubule tracks in cells with partial NIMA function bend and push against the cell tip wall, a phenotype not typical of WT cells (C). See also Movie 5 and 6 in supporting material. (E) Model illustrating the effect of partial inhibition of NIMA on microtubule dynamics. Bars, 5 μm.

The data presented above suggests NIMA-GFP might be locating to the plus ends of microtubules. To test this we generated strains carrying *alcA*-NIMA-GFP in the background of the well-studied microtubule plus end binding protein, EB1 [61], tagged with chRFP (EB1-CR). In *A. nidulans*, EB1-CR has been shown to locate to dynamic comets in the cytoplasm, representing the plus ends of microtubules [62] (Figure 8, Figure 9A, Movie 5 in supporting material). Upon expression of NIMA-GFP, we were able to observe colocalization between NIMA-GFP comets and EB1-CR (Figure 8A, arrows). Live cell imaging revealed the dynamic colocalization of NIMA-GFP and EB1-CR (Figure 8C and Movie 3 in supporting material). We also found that the behavior of EB1 is modified in *alcA*-NIMA expressing cells because EB1-CR exhibits movement both towards and away from the cell tip (Figure 8E) whereas in cells repressed for *alcA*-NIMA expression EB1 only moves unidirectionally towards the cell tip (Figure 8E). Often we were able to track the movement of EB1 comets around cell tips (Figure 8D arrows, Movie 4 in supporting material), very similar to the movement of ectopic NIMA-GFP (Figure 7A, B). These data suggest that in addition to locating to the microtubule plus ends, NIMA might also regulate the function of microtubule plus end binding proteins like EB1 and/or affect microtubule dynamics. Although the dynamic behavior of EB1 at cell tips is modified by ectopic NIMA-GFP, the average rate of EB1 movement did not change significantly (p>0.05) without expression (7.3 μm/min) or with expression of ectopic NIMA-GFP (8.5 μm/min).

EB1 is known to act as an interaction hub at the microtubule plus end since it interacts with, and is responsible for recruiting, several microtubule-plus end binding proteins [63]. To ask whether EB1 is required for the localization of NIMA-GFP to microtubule plus ends, we generated strains expressing ectopic NIMA-GFP in WT cells and EB1-null cells. Strikingly, although 65% (n = 400) of *alcA*-NIMA expressing cells showed distinct NIMA comets, the comet location of NIMA was abolished when EB1 was deleted with less than 2% of the cells showing any NIMA-GFP comets (Figure 8B), indicating that NIMA-GFP localizes to microtubule plus ends in an EB1-dependent manner.

### Partial inactivation of NIMA function modifies microtubule dynamics and EB1 behavior

Our data suggest that NIMA has the potential to regulate microtubule plus-end function. To further investigate this, we generated WT and *nimA7* strains carrying GFP-Tub and EB1-CR, to visualize microtubule tracks and the growing end of microtubules simultaneously. These cells were grown at 35°C, a semi-permissive temperature for *nimA7* that results in partial NIMA function, and observed using live cell time-lapse microscopy with data acquisitions less than one second apart. In WT cells, microtubule tracks with EB1 at the plus ends were readily observed growing towards the cell tip, as denoted by the kymograph in Figure 9A (see also Movie 5 in supporting material). EB1-CR moved predominantly towards the cell tip with an average rate of 15.7+/−4.7 μm/min (n = 63, 6 cells) at this temperature. Under these conditions some *nimA7* cells show cell tip morphology defects, including varying degrees of cell tip swelling and dichotomous cell tips (Figure 3B and C). Notably in such *nimA7* cells with abnormal tips, we detected that EB1 moved not only towards the cell tip as in WT cells, but also in 48% instances where EB1 was tracked (n = 56), EB1 moved away from the cell tip (Figure 9B). Among the population of *nimA7* cells which have more WT-like tip morphology (5 out of 10 cells), EB1 was found to move unidirectionally towards the tip. These data indicate that the misregulation of cell growth and modification of EB1 behavior in *nimA7* cells are linked.

In addition to the alteration of EB1 behavior, *nimA7* cells also show modified microtubule dynamics. In contrast to WT cells, in which microtubules grow towards the tip and then undergo catastrophe and retract away from the tip (Movie 5 in supporting material, Figure 9C), the microtubules in *nimA7* cells can grow towards and then around the cell tip as well as show instances of bending and pushing against the cell tip wall. This phenotype is particularly obvious in the *nimA7* cell shown in Movie 6 in supporting material and Figure 9D, which also shows pronounced cell tip swelling. The data indicate that under conditions where NIMA function is reduced, the dynamics of the microtubule plus end binding protein EB1 can be altered as can the dynamics of microtubules and tip cell growth (Figure 9E).

## Discussion

Using a combination of genetic and cell biological analyses, a previously unrecognized interphase role for the mitotic NIMA kinase in regulating normal cell tip growth in *A. nidulans* hyphae has been uncovered. We present genetic evidence identifying the ESCRT pathway as being required with NIMA for regulating apical cell tip dominance. Further implicating NIMA in regulating normal tip cell growth, functional NIMA-GFP localized to cell tips and some ectopic NIMA-GFP concentrated to the plus ends of interphase microtubules and had the potential to cause marked cell tip growth defects. Importantly, genetic inactivation studies reveal NIMA is required to regulate microtubule plus end binding proteins and microtubule dynamics. This suggests that NIMA has interphase cytoplasmic roles distinct from its established mitotic nuclear functions.

### NIMA functions during cell growth in concert with the ESCRT pathway

Synthetic genetic analysis utilizing deletion of the non-essential Kin3 kinase in budding yeast linked this NIMA-related kinase to two components of the ESCRT pathway. Our subsequent analysis revealed this genetic connection is conserved in *A. nidulans* and extends this to show that NIMA genetically interacts with other ESCRT mutants functioning in distinct steps of the ESCRT pathway. Double mutants that lack ESCRT function in combination with insufficient NIMA show defects in maintaining a single growing tip that are more severe than the single mutants. In ESCRT pathway mutants in budding yeast, *A. nidulans* and mammalian cells, trafficking to the vacuole of cell membrane proteins is impaired [51], [64], [65]. Interestingly, in the absence of ESCRT complex components, there is an accumulation of proteins on the cell membrane in *S. cerevisiae* and higher eukaryotes [42], [66], and in mammalian cells this has also been shown to affect cell polarization [67], [68]. It is therefore possible that the membrane composition and tip cell growth apparatus in *A. nidulans* is altered in ESCRT mutants due to reduced turnover of polarized cell tip membrane localized proteins. These defects, in combination with defects caused by partial inactivation of NIMA at cell tips, and the plus ends of microtubules as discussed below, likely compromise cell tip growth to the point that colony growth is abolished. We therefore suggest that the regulation of the cell tip growth apparatus by NIMA, as well as by turn-over of cell membrane proteins through the ESCRT pathway, jointly ensure normal highly polarized cell tip growth and apical dominance. However, there is accumulating evidence that ESCRT components have also evolved to function in many different cellular processes, including cytokinesis [49] and SPB/centrosome function [69], [70] as recently reviewed [71]. Therefore, it is also possible that the synthetic genetic interactions between NIMA and the ESCRT pathway components might also involve septation and/or SPB defects.

### Novel cytoplasmic locations of NIMA-GFP – At the hyphal cell tip and at the plus ends of microtubules

To further investigate the potential that NIMA might have roles during cell growth as suggested by the genetic interactions with the ESCRT pathway, we employed cell biological approaches. From this analysis, we could readily detect overexpressed NIMA-GFP at the tips of microtubules, but failed to detect endogenously tagged NIMA-GFP at this location, most likely due to its low abundance. In support of this argument, it has been reported that the location of some mammalian microtubule plus tip binding proteins (+TIPs), including EB1, is hard to visualize at microtubule plus ends unless such proteins are ectopically expressed [72], [73]. Lending support to the idea that NIMA-GFP at microtubule tips reflects a physiologically important location, we find that NIMA-GFP locates to the microtubule plus ends in an EB1-dependent manner. EB1 interacts with several microtubule plus end binding proteins and the largest family has a consensus sequence, (S/T)X(I/L)P, amidst a region rich in basic residues [72], [74]. We find that NIMA contains two consensus motifs, SRLP (amino acids: 563–566) as well as SKIP (amino acids: 586–589), which represent potential binding sites for EB1. Phosphorylation by CDK1 and GSK3 at residues near the (S/T)X(I/L)P motif of the human +TIP, CLASP2, disrupts its interaction with EB1 during mitosis [75]–[77]. Intriguingly, NIMA also has potential CDK1 phosphorylation ‘SP’ sites near its (S/T)X(I/L)P consensus motifs and has been shown to be an effective *in vitro* substrate of CDK1 [29]. This suggests a potential model wherein during mitosis CDK1 phosphorylation of NIMA might modify its functions at cytoplasmic microtubule plus ends, and perhaps at cell tips, promoting the disassembly of cytoplasmic microtubules as a prelude to mitotic spindle formation.

The localization of some endogenously tagged NIMA-GFP to cell tips is detectable in fast growing hyphal cells. Paradoxically, upon overexpression we failed to detect NIMA-GFP at cell tips even though extra NIMA-GFP profoundly affected cell tip growth. It is possible that the modifications of the cell tip apparatus caused by extra NIMA-GFP renders its normal NIMA targeting properties ineffective. Of note, several other +TIPs have been reported to locate to an intracellular structure (in addition to their location to microtubule tips) such as the plasma membrane (CLASPs, ACF7) or the ER (STIM1), where they are proposed to help regulate microtubules in a spatially-controlled manner [72], [76], [78]–[80]. We postulate that the location of NIMA-GFP to both microtubule plus ends as well as to the cell tip region may reflect a similar role for NIMA in regulating microtubules dynamics at the cell apex. It is known that cytoplasmic microtubules that grow towards the cell apex undergo catastrophe after they make contact with the cell tip in *A. nidulans* [60], [81], [82]. Cell-tip localized NIMA, in combination with NIMA at microtubule ends, could potentially contribute to regulating this switch in microtubule dynamics. If this were true, when NIMA function is impaired microtubules might continue to grow after reaching the cell tip. Consistent with this expectation, we find that in cells with partial NIMA function, microtubules show an increased tendency to bend and grow around the cell tip, which in turn might cause the mis-targeting of polarity factors at the cell apex. There is precedence for this idea from work in *S. pombe*. It has been shown that promoting the contact of microtubule plus ends with sites on the plasma membrane other than the growing cell tip in *tea1* mutants, by artificially changing the shape of *S. pombe* cells, can promote mislocalization of polarity factors in a manner dependent on the EB1 orthologue, Mal3, causing defects in polarized growth [83].

### Conserved functions for NIMA in interphase and mitosis

Our data provides the first evidence to support the idea proposed in a recent review that NIMA kinases might have interphase functions involving the regulation of microtubule plus end binding proteins [25]. However, our studies are not the first to implicate a NIMA kinase in regulating interphase microtubules. Indeed, recent studies propose a role for Nek7 in regulating microtubule dynamics in HeLa cells and mouse embryonic fibroblasts, and for Nek3 in regulating microtubule acetylation in highly polarized neurons [84], [85]. Of particular interest are the parallels between the roles NIMA plays in regulating microtubules and cell wall growth in *A. nidulans* and the similar regulatory relationships seen between plant NIMA related kinases 6, 4 and 5 and plant cell wall growth and microtubule dynamics [86], [87]. The *Arabidopsis thaliana* NIMA-related kinase Nek6 locates to cortical microtubules, regulates cell wall growth, and phosphorylates β-tubulin in vitro. It might therefore be interesting to explore the potential that the plant ESCRT pathway, as in *A. nidulans*, might affect plant cell wall development in combination with Nek kinases. Similarly, mammalian Nek6 and Nek7 can phosphorylate tubulin *in vitro* [88] and multiple NIMA related kinases, including Nek8, Nek1, Nek7 and Nek2, have been implicated in regulating the growth and function of cilia [25], [26]. One additional possibility, supported by our data, is that Neks also have the potential to regulate ciliary functions through an effect on microtubule plus ends. Therefore further exploration of the interphase function of NIMA involving the regulation of cytoplasmic microtubules and cell growth will be important to obtain a more complete understanding of the functions of NIMA related kinases.

Finally, results from *S. cerevisiae* have described a role for the Cdk1 cell cycle kinase in cell growth through the regulation of membrane trafficking and the organization of zones for endocytosis and exocytosis [89], [90]. Our studies therefore add further support to the concept that cell cycle regulatory kinases can also regulate cell growth processes, providing a regulatory system to coordinate cell cycle progression and mitosis with polarized cell surface growth. Such regulation also likely involves protein dephosphorylation as the *A. nidulans* BIMG protein phosphatase 1 locates to the hyphal cell tip [91], suggesting that fungal cell growth could be regulated by the antagonistic phosphorylation-dephosphorylation activities of cell cycle kinases and phosphatases ensuring integration of mitotic nuclear divisions with polarized cell tip growth.

## Materials and Methods

Genome and protein sequences were obtained from the Aspergillus Genome Database (http://aspgd.org/) or the Aspergillus Comparative Database at the Broad Institute (http://www.broadinstitute.org/annotation/genome/aspergillus_group/MultiHome.html). BLAST searches were performed at http://aspgd.org/ or at http://blast.ncbi.nlm.nih.gov/Blast.cgi. Sequences were viewed and mapped for open reading frames on Gene Runner Version 3.05 (Hastings Software Inc.). Standard conditions were used for the generation and propagation of *A. nidulans* strains as described in [92] with minor alterations. The genotypes of strains used in this study are provided in supporting material Table S1. Tagging of genes with fluorescent probes at the endogenous locus, gene deletions and heterokaryon rescue analysis were done as previously described [50], [93], [94]. For live cell imaging, conidiospores were grown in liquid media containing glucose in 35 mm glass bottom petri dishes (MatTek Cultureware, Ashland, MA, USA). Cells were observed using a 60× 1.49 NA TIRF objective lens on an Eclipse TE 2000-U (Nikon, Inc.) microscope equipped with an UltraView ERS spinning disk confocal system or using an UltraVIEW Vox CSUX1 system (PerkinElmer Inc.). Images were captured using an ORCA-AG camera or an EMCCD camera (Hamamatsu C9100-13). The expression of genes regulated by the *alcA* promoter [57] was controlled by using different carbon sources – either 55 mM glucose (repressed), 4.6 ml/L glycerol (derepressed) or 1% ethanol (induced) in minimal media, or 100 mM threonine (induced) in lactose yeast extract containing medium. 2.4 μg/mL benomyl was used to depolymerize microtubules and the actin depolymerizing drug, latrunculin B was used at a concentration of 40 μg/mL [5], [55]. Long hyphal cells were obtained using an agar-anchor method, in which conidiospores were inoculated at a single point on the culture dish, allowed to dry and then anchored using 50 μL of molten agar containing complete media. The agar was allowed to solidify and then liquid media was gently added to the dish. Temperature controlled experiments were carried out using Delta T culture dishes and heating equipment from Bioptechs, Inc., PA, USA. When fixing cells for microscopy, conidiospores were germinated in yeast extract glucose liquid media on coverslips. DAPI stained fixed cells were observed using an E800 microscope (Nikon, Inc.) and an UltraPix digital camera (Life Science Resources, Ltd). Movies and images were analyzed using UltraView, ImageJ or Volocity software and kymographs were generated using ImageJ, version 1.46 m (http://rsbweb.nih.gov/ij/). The rate of movement was calculated from the slopes of the movement trace from kymographs.

## Supporting Information

Figure S1 *nimA7* does not exhibit genetic interaction with 7 of the 10 *A. nidulans* orthologues of *S. cerevisiae* genes that interact with *kin3* (also Table 1). The images show colonies grown at 35°C, a semi-permissive temperature of *nimA7*, from spores of indicated genotypes. Strains: WT  =  R153, *nimA7*  =  MG44, *ΔAn-zuo1*  =  MG8, *nimA7 + ΔAn-zuo1*  =  MG61, *ΔAn-bud14*  =  MG19, *nimA7* + *ΔAn-bud14*  =  MG67, *ΔAn-ssz1*  =  MG10, *nimA7* + *ΔAn-ssz1*  =  MG62, *ΔAn-dot1*  =  MG12, *nimA7* + *ΔAn-dot1*  =  MG63, *ΔAn-mph1*  =  MG22, *nimA7* + *ΔAn-mph1  = * MG68, *ΔAn-hsl7  = * MG14 and *nimA7* + *ΔAn-hsl7  = * MG64.
(PDF)

Figure S2 (A) Deletion of *An-vps23* leads to the formation of heterokaryons, analyzed here by the heterokaryon rescue technique. Growth of conidia isolated from WT, *pyrG^−^* and heterokaryons formed following *An-vps23* deletion on non-selective and selective media shows that the deletion of *An-vps23* severely impairs growth. (B) Diagnostic PCR confirms the presence of *An-vps23* WT and *An-vps23*-deleted nuclei in the heterokaryons.
(PDF)

Figure S3 Spontaneous *Δvps36* suppressor mutations can suppress the interaction between *nimA7* and *Δvps36.* Conidia isolated from *nimA7+Δvps36* heterokaryons were allowed to form colonies at the permissive temperature (32°C). After 5 days, the formation of suppressor colonies were seen, similar to colonies marked with arrows in (A). Conidia from two different suppressor colonies were isolated and spread on plates and allowed to grow either at permissive or semi-permissive temperatures (35°C). The data shows that although *nimA7+Δvps36* are unable to form colonies at this temperature (A) *nimA7+Δvps36* colonies that also carry suppressor mutations are able to do so (B and C).
(PDF)

Figure S4 (A) The cell tip location of NIMA is unchanged in the absence of ESCRT complex function. NIMA-GFP is detectable at 28% of WT cell tips (n = 117; strain KF005) and a comparable 31% of *ΔAn-vps23* (n = 129; strain MGH61) cell tips at 35°C. (B) NIMA-GFP levels at the cell tip decrease in mitosis when NIMA displays its characteristic nuclear location. Bar, 5 μm.
(PDF)

Figure S5 Colony growth of strains expressing ectopic NIMA constructs. (A) Growth of the indicated strains carrying *alcA* driven NIMA constructs under conditions when ectopic NIMA is not expressed (lactose) or is expressed (threonine) compared to WT. (B) Growth of a strain carrying *alcA*-NIMA-GFP when NIMA-GFP is not expressed (Glucose) and when it is expressed (Glycerol) compared to WT. Strains: WT  =  R153, *alcA*-NIMA-RegD-GFP  =  CDS131, *alcA*-NIMA-GFP  =  CDS683.
(PDF)

Movie S1 Dome-shaped localization of NIMA-GFP at the cell tip. Delay  =  30 s. Frame rate  =  15 fps. Length of movie: 25 min.
(AVI)

Movie S2 Cytoplasmic movement of ectopic NIMA-GFP comets. Delay  =  5.8 s. Play rate  =  15 fps. Length of Movie  =  10.6 min.
(AVI)

Movie S3 Colocalization of ectopic NIMA-GFP with EB1-CR. Delay  = 11.5 s. Play rate  =  3 fps. Length of movie  =  2 min.
(AVI)

Movie S4 Movement of EB1-CR under conditions allowing expression of *alcA*-NIMA-GFP (glycerol as carbon source). Delay  =  1 s. Play rate  =  15 fps. Length of movie  =  2 min.
(AVI)

Movie S5 Dynamics of EB1-CR and Mts in a Wt cell at 35°C. Delay  =  0.86 s. Play rate 30 fps. Length of movie  =  3.3 min.
(AVI)

Movie S6 EB1-CR and Mt dynamics in a *nimA7* cell at 35°C. Delay  =  0.81 s. Play rate  =  30 fps. Length of movie  =  7 min.
(AVI)

Table S1 Genotypes of strains used in the study.
(PDF)
