# Supplementary figures and images for: Identification of Interphase Functions for the NIMA Kinase Involving Microtubules and the ESCRT Pathway
Source: PLoS Genet. 2014 Mar 27;10(3):e1004248. doi: 10.1371/journal.pgen.1004248 (PMC3967960; doi:10.1371/journal.pgen.1004248)

Figure S1

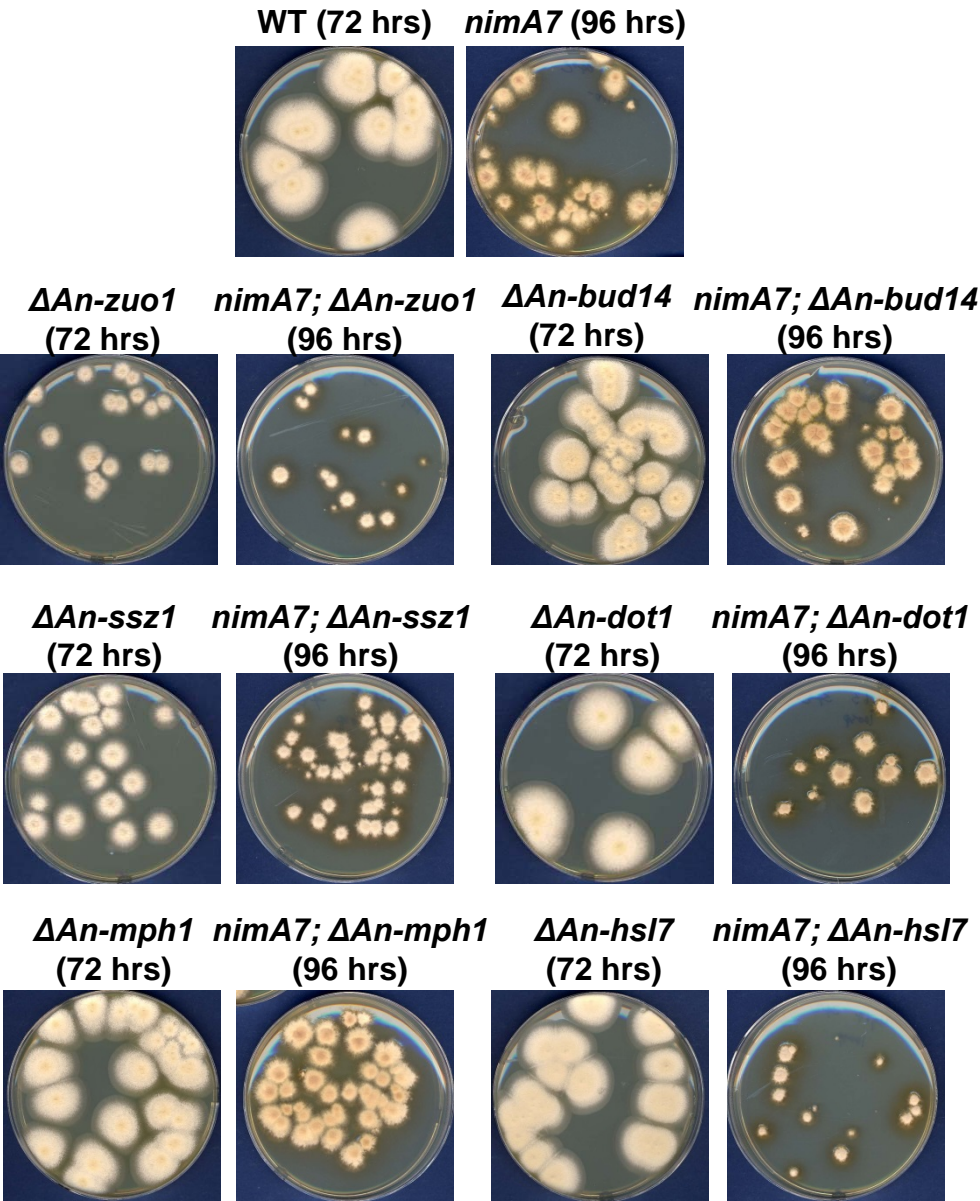

Supplement: Figure S1 — nimA7 does not exhibit genetic interaction with 7 of the 10 A. nidulans orthologues of S. cerevisiae genes that interact with kin3 (also Table 1). The images show colonies grown at 35°C, a semi-permissive temperature of nimA7, from spores of indicated genotypes. Strains: WT = R153, nimA7 = MG44, ΔAn-zuo1 = MG8, nimA7 + ΔAn-zuo1 = MG61, ΔAn-bud14 = MG19, nimA7 + ΔAn-bud14 = MG67, ΔAn-ssz1 = MG10, nimA7 + ΔAn-ssz1 = MG62, ΔAn-dot1 = MG12, nimA7 + ΔAn-dot1 = MG63, ΔAn-mph1 = MG22, nimA7 + ΔAn-mph1 = MG68, ΔAn-hsl7 = MG14 and nimA7 + ΔAn-hsl7 = MG64. (PDF) [file pgen.1004248.s001.pdf]

Figure S2

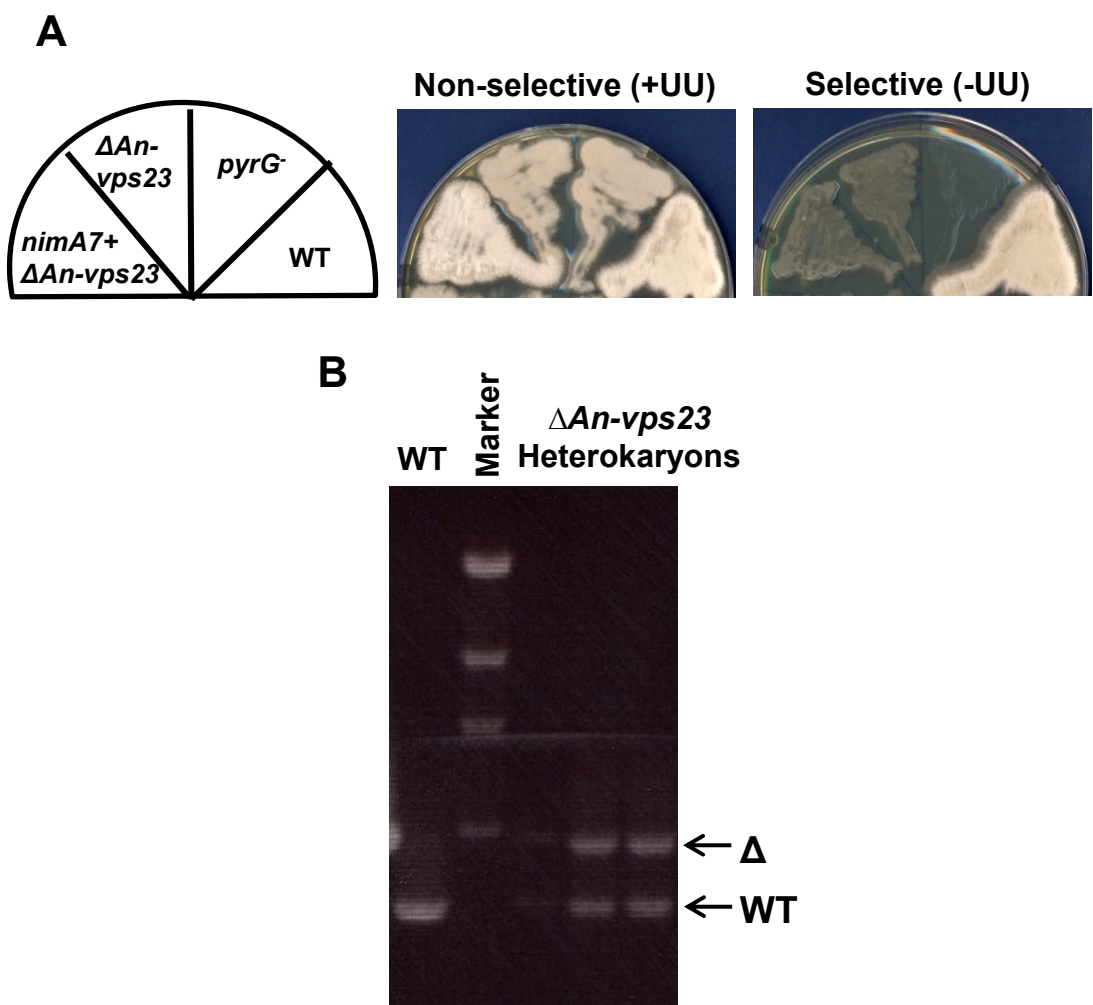

Supplement: Figure S2 — (A) Deletion of An-vps23 leads to the formation of heterokaryons, analyzed here by the heterokaryon rescue technique. Growth of conidia isolated from WT, pyrG− and heterokaryons formed following An-vps23 deletion on non-selective and selective media shows that the deletion of An-vps23 severely impairs growth. (B) Diagnostic PCR confirms the presence of An-vps23 WT and An-vps23-deleted nuclei in the heterokaryons. (PDF) [file pgen.1004248.s002.pdf]

Figure S3

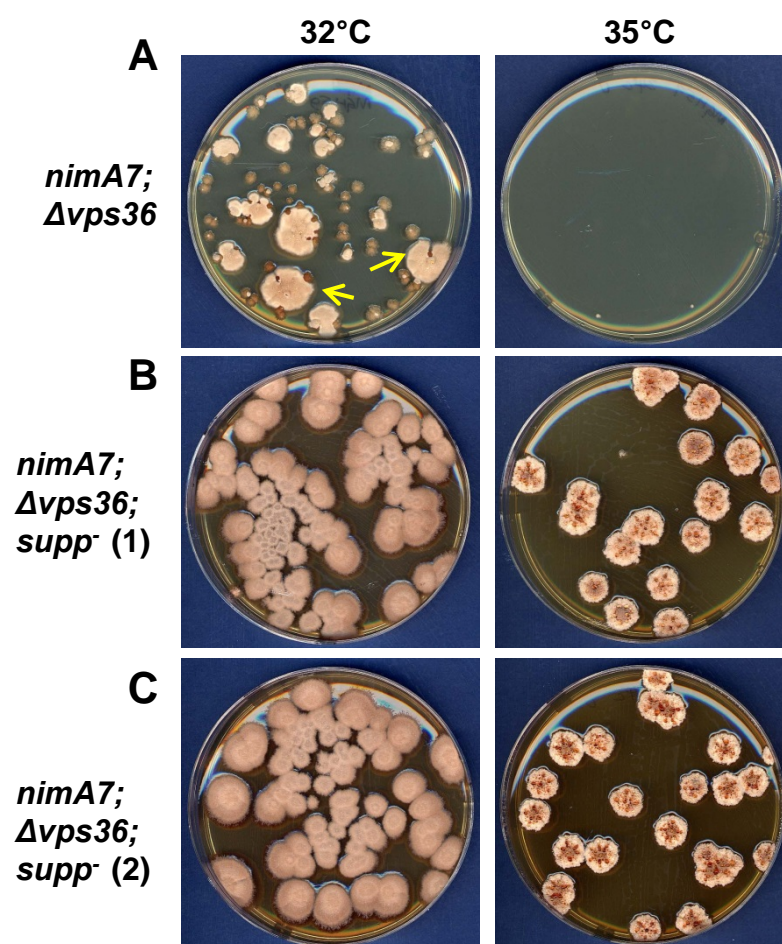

Supplement: Figure S3 — Spontaneous Δvps36 suppressor mutations can suppress the interaction between nimA7 and Δvps36. Conidia isolated from nimA7+Δvps36 heterokaryons were allowed to form colonies at the permissive temperature (32°C). After 5 days, the formation of suppressor colonies were seen, similar to colonies marked with arrows in (A). Conidia from two different suppressor colonies were isolated and spread on plates and allowed to grow either at permissive or semi-permissive temperatures (35°C). The data shows that although nimA7+Δvps36 are unable to form colonies at this temperature (A) nimA7+Δvps36 colonies that also carry suppressor mutations are able to do so (B and C). (PDF) [file pgen.1004248.s003.pdf]

**Figure S4**

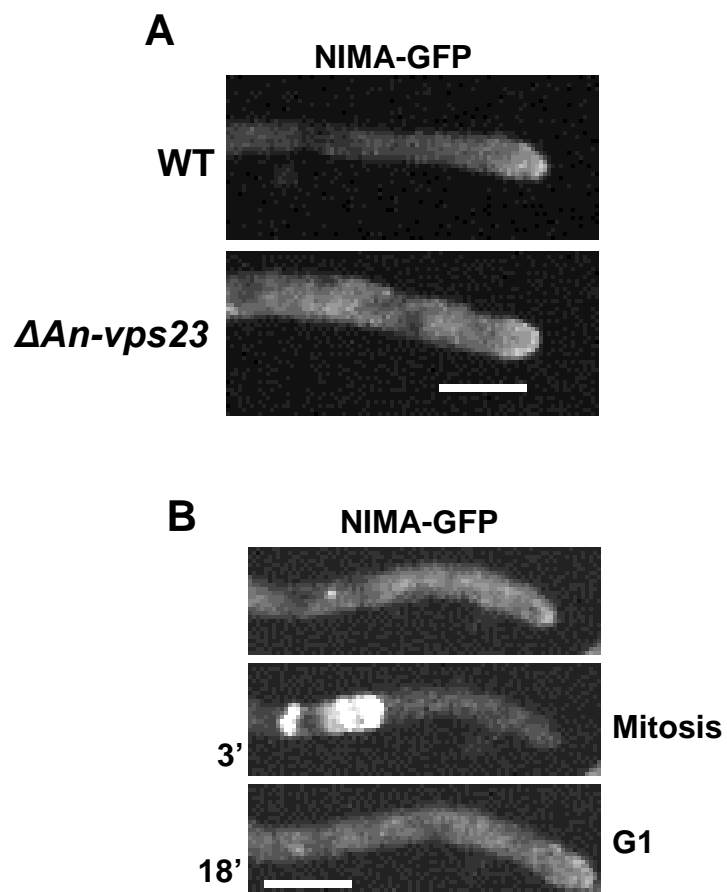

Supplement: Figure S4 — (A) The cell tip location of NIMA is unchanged in the absence of ESCRT complex function. NIMA-GFP is detectable at 28% of WT cell tips (n = 117; strain KF005) and a comparable 31% of ΔAn-vps23 (n = 129; strain MGH61) cell tips at 35°C. (B) NIMA-GFP levels at the cell tip decrease in mitosis when NIMA displays its characteristic nuclear location. Bar, 5 μm. (PDF) [file pgen.1004248.s004.pdf]

Figure S5

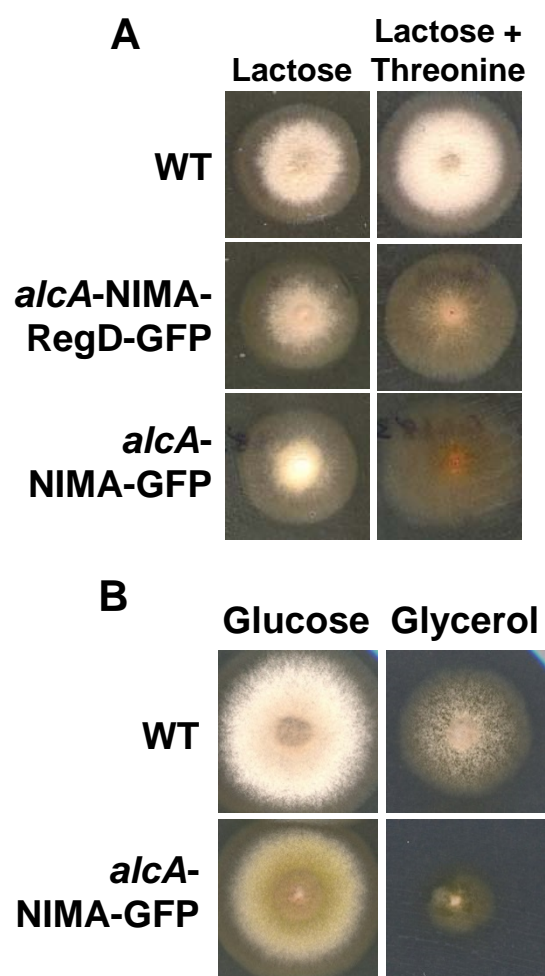

Supplement: Figure S5 — Colony growth of strains expressing ectopic NIMA constructs. (A) Growth of the indicated strains carrying alcA driven NIMA constructs under conditions when ectopic NIMA is not expressed (lactose) or is expressed (threonine) compared to WT. (B) Growth of a strain carrying alcA-NIMA-GFP when NIMA-GFP is not expressed (Glucose) and when it is expressed (Glycerol) compared to WT. Strains: WT = R153, alcA-NIMA-RegD-GFP = CDS131, alcA-NIMA-GFP = CDS683. (PDF) [file pgen.1004248.s005.pdf]
