# Supplementary material for: Identification of Interphase Functions for the NIMA Kinase Involving Microtubules and the ESCRT Pathway
Source: PLoS Genet. 2014 Mar 27;10(3):e1004248. doi: 10.1371/journal.pgen.1004248 (PMC3967960; doi:10.1371/journal.pgen.1004248)
Supplement: Table S1 — Genotypes of strains used in the study. (PDF) [file pgen.1004248.s012.pdf]

Table S1: List of strains used in the study

**Name**	**Genotype** (all strains also carry *veA1*)
KF45	*nimA-GFP::pyrG*^{AF}*; NDC80-CR::pyroA*^{AF}*; argB2; nirA14?; sE15?; wA3*
CDS683	*alcA::NIMA-GFP::pyr4*$^{+}$ *(pyrG89); Nup49-CR::pyroA*^{AF}*; chaA1 (pyroA4? nirA14?)*
CDS1068	*GFP-S-tag::pyrG*^{AF}*(pyrG89); ΔnKuA*^{Ku70}*::argB(argB2); pyroA4; nirA14; sE15, wA3; fwA1; chaA1*
CDS131	*alcA::*$^{HA\text{-}285}$*ΔNnimA-GFP::pyr4 (pyrG89); pyr4*$^{+}$*; pyroA4; wA*
R153	*pyroA4*; *wA3*
MG385	*alcA::NIMA-GFP::pyr4*$^{+}$ *(pyrG89); EB1-CR::pyro*^{AF} *(pyroA4?); ΔnKuA*^{Ku70}*::argB/ argB*$^{+}$*?, wA3*
MG409	*alcA::NIMA-GFP::pyr4*$^{+}$ *(pyrG89); argB2; sE15; wA3*
MG410	*alcA::NIMA-GFP::pyr4*$^{+}$*; pyrG89; ΔAn-EB1::pyrG*^{AF}*(pyrG89); argB2; sE15; wA3*
MG395	*GFP-tubA; EB1-CR::pyro*^{AF} *(pyroA4); argB2; sE15; nirA14?*
MG397	*nimA7; GFP-tubA; EB1-CR::pyro*^{AF} *(pyroA4); argB2; sE15; nirA14?*
MG44	*nimA7; ΔnKuA*^{Ku70}*::argB (argB2); riboA1; pyroA4; pyrG89; nicB8/A2?; wA3*
MG8	*ΔAn-zuo1::pyrG*^{AF} *(pyrG89); ΔnKuA*^{Ku70}*::argB (argB2); pyroA4; nirA14; sE15, wA3; fwA1; chaA1*
MG61	*nimA7; ΔAn-zuo1::pyrG*^{AF} *(pyrG89); ΔnKuA*^{Ku70}*::argB (argB2); riboA1; pyroA4; nicB8/A2?; wA3*
MG19	*ΔAn-bud14::pyrG*^{AF} *(pyrG89); ΔnKuA*^{Ku70}*::argB(argB2); pyroA4; nirA14; sE15, wA3; fwA1; chaA1*
MG67	*nimA7; ΔAn-bud14::pyrG*^{AF} *(pyrG89); ΔnKuA*^{Ku70}*::argB (argB2); riboA1; pyroA4; nicB8/A2?; wA3*
MG10	*ΔAn-ssz1::pyrG*^{AF} *(pyrG89); ΔnKuA*^{Ku70}*::argB(argB2); pyroA4; nirA14; sE15, wA3; fwA1; chaA1*
MG62	*nimA7; ΔAn-ssz1::pyrG*^{AF} *(pyrG89); ΔnKuA*^{Ku70}*::argB (argB2); riboA1; pyroA4; nicB8/A2?; wA3*
MG12	*ΔAn-dot1::pyrG*^{AF} *(pyrG89); ΔnKuA*^{Ku70}*::argB(argB2); pyroA4; nirA14;*

	sE15, wA3; fwA1; chaA1
MG63	*nimA7; ΔAn-dot1::pyrG*^{AF} *(pyrG89); ΔnKuA*^{Ku70}*::argB (argB2); riboA1; pyroA4; nicB8/A2?; wA3*
MG22	*ΔAn-mph1::pyrG*^{AF} *(pyrG89); ΔnKuA*^{Ku70}*::argB(argB2); pyroA4; nirA14; sE15, wA3; fwA1; chaA1*
MG68	*nimA7; ΔAn-mph1::pyrG*^{AF} *(pyrG89); ΔnKuA*^{Ku70}*::argB (argB2); riboA1; pyroA4; nicB8/A2?; wA3*
MG14	*ΔAn-hsl7::pyrG*^{AF} *(pyrG89); ΔnKuA*^{Ku70}*::argB(argB2); pyroA4; nirA14; sE15, wA3; fwA1; chaA1*
MG64	*nimA7; ΔAn-hsl7::pyrG*^{AF} *(pyrG89); ΔnKuA*^{Ku70}*::argB (argB2); riboA1; pyroA4; nicB8/A2?; wA3*
SO451	*pyrG89; ΔnKuA*^{Ku70}*::argB(argB2); pyroA4; nirA14; sE15, wA3; fwA1; chaA1*
MGH21	Heterokaryon carrying both WT *An-vps23* and *ΔAn-vps23::pyrG*^{AF} *(pyrG89); ΔnKuA*^{Ku70}*::argB(argB2); pyroA4; nirA14; sE15, wA3; fwA1; chaA1*
MGH26	Heterokaryon carrying both WT *An-vps25* and *ΔAn-vps25::pyrG*^{AF} *(pyrG89); ΔnKuA*^{Ku70}*::argB(argB2); pyroA4; nirA14; sE15, wA3; fwA1; chaA1*
MGH19	Heterokaryon carrying both WT *An-vps23* and *ΔAn-vps23::pyrG*^{AF} *(pyrG89); nimA7; ΔnKuA*^{Ku70}*::argB (argB2); riboA1; pyroA4; nicB8/A2?; wA3*
MGH14	Heterokaryon carrying both WT *An-vps25* and *ΔAn-vps25::pyrG*^{AF} *(pyrG89); nimA7; ΔnKuA*^{Ku70}*::argB (argB2); riboA1; pyroA4; nicB8/A2?; wA3*
MGH17	Heterokaryon carrying both WT *An-vps23* and *ΔAn-vps23::pyrG*^{AF} *(pyrG89); nimA7; ΔnKuA*^{Ku70}*::argB (argB2); riboA1; pyroA4; nicB8/A2?; wA3*
MG71	*nimA7; pyrG*^{AF}*insertion (pyrG89); ΔnKuA*^{Ku70}*::argB (argB2); riboA1; pyroA4; nicB8/A2?; wA3*
MGH53	Heterokaryon carrying both WT *An-vps28* and *ΔAn-vps28::pyrG*^{AF} *(pyrG89); ΔnKuA*^{Ku70}*::argB(argB2); pyroA4; nirA14; sE15, wA3; fwA1; chaA1*
MGH55	Heterokaryon carrying both WT *An-vps28* and *ΔAn-vps28::pyrG*^{AF} *(pyrG89); nimA7; ΔnKuA*^{Ku70}*::argB (argB2); riboA1; pyroA4; nicB8/A2?;*

	wA3
MGH49	Heterokaryon carrying both WT *An-vps24* and *ΔAn-vps24::pyrG*^{AF} *(pyrG89); ΔnKuA*^{Ku70}*::argB(argB2); pyroA4; nirA14; sE15, wA3; fwA1; chaA1*
MGH51	Heterokaryon carrying both WT *An-vps24* and *ΔAn-vps24::pyrG*^{AF} *(pyrG89); nimA7; ΔnKuA*^{Ku70}*::argB (argB2); riboA1; pyroA4; nicB8/A2?; wA3*
MGH57	Heterokaryon carrying both WT *An-vps36* and *ΔAn-vps36::pyrG*^{AF} *(pyrG89); ΔnKuA*^{Ku70}*::argB(argB2); pyroA4; nirA14; sE15, wA3; fwA1; chaA1*
MGH59	Heterokaryon carrying both WT *An-vps36* and *ΔAn-vps36::pyrG*^{AF} *(pyrG89); nimA7; ΔnKuA*^{Ku70}*::argB (argB2); riboA1; pyroA4; nicB8/A2?; wA3*
MGH45	Heterokaryon carrying both WT *An-vps4* and *ΔAn-vps4::pyrG*^{AF} *(pyrG89); ΔnKuA*^{Ku70}*::argB(argB2); pyroA4; nirA14; sE15, wA3; fwA1; chaA1*
MGH47	Heterokaryon carrying both WT *An-vps4* and *ΔAn-vps4::pyrG*^{AF} *(pyrG89); nimA7; ΔnKuA*^{Ku70}*::argB (argB2); riboA1; pyroA4; nicB8/A2?; wA3*

? Indicates marker might be present but covered by another mutation or the allele present not known.
